# Highly Ordered Thermoplastic Polyurethane/Aramid Nanofiber Conductive Foams Modulated by Kevlar Polyanion for Piezoresistive Sensing and Electromagnetic Interference Shielding

**DOI:** 10.1007/s40820-023-01062-0

**Published:** 2023-04-07

**Authors:** Kunpeng Qian, Jianyu Zhou, Miao Miao, Hongmin Wu, Sineenat Thaiboonrod, Jianhui Fang, Xin Feng

**Affiliations:** 1 School of Materials Sciences and Engineering, Shanghai University, Shanghai, 200444 People’s Republic of China; 2 Research Center of Nano Science and Technology, Shanghai University, Shanghai, 200444 People’s Republic of China; 3 National Nanotechnology Center (NANOTEC), National Science and Technology Development Agency (NSTDA), Thailand Science Park, Pathum Thani, 12120 Thailand

**Keywords:** Highly ordered conductive foams, MXene, Nanofiber, Thermoplastic, Kevlar polyanion, Piezoresistive sensing, Electromagnetic interference shielding

## Abstract

**Abstract:**

Highly ordered and uniformly porous structure of conductive foams is a vital issue for various functional purposes such as piezoresistive sensing and electromagnetic interference (EMI) shielding. With the aids of Kevlar polyanionic chains, thermoplastic polyurethane (TPU) foams reinforced by aramid nanofibers (ANF) with adjustable pore-size distribution were successfully obtained via a non-solvent-induced phase separation. In this regard, the most outstanding result is the in situ formation of ANF in TPU foams after protonation of Kevlar polyanion during the NIPS process. Furthermore, in situ growth of copper nanoparticles (Cu NPs) on TPU/ANF foams was performed according to the electroless deposition by using the tiny amount of pre-blended Ti_3_C_2_T_x_ MXene as reducing agents. Particularly, the existence of Cu NPs layers significantly promoted the storage modulus in 2,932% increments, and the well-designed TPU/ANF/Ti_3_C_2_T_x_ MXene (PAM-Cu) composite foams showed distinguished compressive cycle stability. Taking virtues of the highly ordered and elastic porous architectures, the PAM-Cu foams were utilized as piezoresistive sensor exhibiting board compressive interval of 0–344.5 kPa (50% strain) with good sensitivity at 0.46 kPa^−1^. Meanwhile, the PAM-Cu foams displayed remarkable EMI shielding effectiveness at 79.09 dB in X band. This work provides an ideal strategy to fabricate highly ordered TPU foams with outstanding elastic recovery and excellent EMI shielding performance, which can be used as a promising candidate in integration of satisfactory piezoresistive sensor and EMI shielding applications for human–machine interfaces.

**Graphical Abstract:**

**Supplementary Information:**

The online version contains supplementary material available at 10.1007/s40820-023-01062-0.

## Highlights

- Kevlar polyanionic chains induced the formation of highly ordered porous thermoplastic polyurethane foams with adjustable pore sizes.
- A tiny amount of Ti_3_C_2_T_x_ MXene was performed as reducing agent for the electroless deposition of copper nanoparticles.
- The conductive foam reinforced by aramid nanofibers exhibited excellent piezoresistive sensing and electromagnetic interference shielding performance.

**Supplementary Information:**

The online version contains supplementary material available at 10.1007/s40820-023-01062-0.

## Graphical abstract

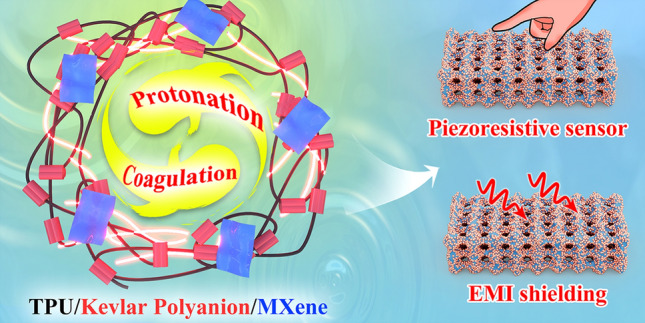

## Introduction

With the rapid development of integrated electronic equipment in 5G era, the increasing demand of multifunctional materials with lightweight, high conductivity and flexible/wearable properties has drawn an extensive interest worldwide [1–5]. To date, the rational design of conductive foams/aerogels/sponges composed of elastic polymer substrates and conductive fillers with improved mechanical and electrical properties is becoming a research hot spot, since the porous structure with conductive networks would lead to the change in contact resistance for strain sensors or increase the multiple reflection and absorption dissipation of incident electromagnetic waves (EMWs) by the enhancement of interface polarization [6–8].

As a typical segmented copolymer composed of hard segments and soft segments, thermoplastic polyurethane (TPU) has been demonstrated as an ideal substrate for porous composites with remarkable elastic resilience and tensile property [9, 10]. For the demand of porous structure, many chemical foaming agents were introduced to fabricate TPU-based porous materials such as butane [11], azodicarbonamide and sodium bicarbonate [12], but the inherent toxicity limits their wide applications. On the other hand, the physical pore-forming methods such as freeze-drying and supercritical carbon dioxide (scCO_2_) foaming are environment friendly for TPU-based porous materials [13–15]. However, the reaction conditions for scCO_2_ like high temperature and high pressure in a sealed vessel should be strictly kept, with yet the resulted porous materials presenting pore sizes ranging from few micrometers to hundreds of micrometers. To achieve orderly regular porous structures, directional freeze-drying techniques were elaborately designed to control the pore sizes, alignment and cellular configuration by adjusting the frozen conditions within complicated vessels [16]. Recently, phase separation technique has been developed as a simple and environmentally friendly method to prepare TPU-based porous materials [17, 18]. During the non-solvent-induced phase separation (NIPS) process, TPU with a layer of polymer solution immersed in a non-solvent bath, resulting in phase separation with polymer-rich and polymer-poor phases caused by thermodynamic instability [19, 20]. After the continuously exchanging between solvent and non-solvent in the coagulation bath, the porous architectures were accordingly obtained by the solidification of polymer-rich phases and pore-forming of polymer-poor phases, respectively [21, 22]. Usually, porous TPU materials by NIPS displayed an asymmetrical structure composed of spongy-shaped macrovoids or finger-like structures due to the slow permeation of non-solvent (water) into the bottom of TPU solution [21, 23–25]. Polymerizable ionic liquid copolymer (PIL) containing anion-cation pairs was successfully utilized to decrease the surface tension gradient at the non-solvent phase surface, to decrease the mass-transfer and the size of solvent dilute phase. Consequently, homogeneous microcellular structure of TPU/carbon nanotubes (CNTs)/PIL composite foams with small cell size for high-performance EMI shielding was obtained [17].

Interestingly, Kevlar polyanionic chains have also been proved to possess the ability of being integrated with other materials via hydrogen bonding interactions. Kevlar polyanion was obtained from aramid pulp, commonly known as Kevlar fibers [26]. By the solvent exchange of poly (vinyl alcohol) (PVA) and Kevlar polyanion solution in dimethylsulfoxide (DMSO), aramid nanofibers (ANF) were achieved after the protonation process of Kevlar polyanion and subsequently combined with PVA via hydrogen bonding, leading to the remarkable enhancement of mechanical properties for the resultant ANF/PVA composites [27, 28]. It is expected that ANF retains the distinguished advantages of Kevlar fibers such as high mechanical strength and high-temperature stability. Thanks to the similar molecular structures of ANF with waterborne polyurethanes (PU), the formation of multiple interactions between amide-containing PU and ANF was accordingly confirmed, which is beneficial to the mechanical improvement of ANF/PU nanocomposites [29].

To effectively utilize porous structure for the highly conductive foams/aerogels/sponges, the dip coating or impregnation of conductive slurries was always adopted [30, 31]. Ti_3_C_2_T_x_ MXene has attracted widespread attention owing to its incomparable metal conductivity. Particularly, the rich hydrophilic functional groups (–OH, = O, and –F) on the surface of MXene provide tremendous potential for strong adhesion with other materials [32–37]. Wang et al. inserted carbon nanotubes (CNTs)/MXene composites into a multi-channel 3D cellulose scaffold (CS) via vacuum impregnation and then wrapped with poly (dimethylsiloxane) (PDMS) to fabricate a wood-based piezoresistive sensor with high EMI shielding effect [38]. Weng et al. fabricated silver nanowire (AgNW)/MXene hybrid sponges using the commercial melamine formaldehyde as the templates by a combination of dip-coating and unidirectional freeze-drying methods [39]. However, the performance of the conductive porous composites is closely related to the dip coating time or impregnation cycles in some extent. Most notably, by virtue of the susceptible oxidization of Ti_3_C_2_T_x_ MXene in humid or aqueous environment conditions [40, 41], an in situ hybridization of Ag, Au and Pd nanoparticles with Ti_3_C_2_T_x_ MXene was developed by using delaminated MXene as reduction/nucleation sites without external reducing agents [42]. Moreover, MXene with abundant surface terminations served as a platform for in situ and spontaneous reduction of AuCl_4_¯, leading to the formation of Au nanostructures on MXene for multifunctional sensing and energy harvesting [43]. Therefore, the surface metallization of Ti_3_C_2_T_x_ MXene by the *in situ* reduction of metal nanoparticles to form continuously conductive network is becoming a significant and effective method.

In this work, Kevlar polyanionic chains induced highly ordered porous TPU/ANF/MXene (PAM) foams were successfully assembled via a NIPS process, and the *in situ* reduction of copper nanoparticles (Cu NPs) layers wrapped on PAM foams (PAM-Cu) was achieved through electroless deposition (ELD) method using small amounts of Ti_3_C_2_T_x_ MXene as reduction agents, without the need of traditional complicated sensitization and activation processes. After the introduction of Kevlar polyanion into TPU/DMSO solution, the fluidity increased remarkably, resulting in a moderate molecular-to-lamellar-level ordering disruption. The melting enthalpy of hard domains was accordingly decreased, thereby attenuating the Marangoni convection to form homogeneously dispersed pores. Furthermore, Kevlar polyanion was simultaneously transferred into ANF during the protonation process. Consequently, the electrically conductive PAM-Cu foams with outstanding elastic recovery ability displayed excellent EMI shielding performance. More importantly, the PAM-Cu foams were also applied as ideal piezoresistive sensors, manifesting great fatigue resistance, which can be used to detect various human activities in real time. Certainly, the Kevlar polyanion-induced assembly strategy is expected to solve the problem of asymmetrical structure of porous composites, to control the pore size of TPU-based conductive foams effectively.

## Experimental Sections

### Materials

Thermoplastic polyurethane (TPU, 1185A) granule was obtained from BASF Co., Ltd. Aramid pulp was brought from Zhongli New Material Technology Co., Ltd. Ti_3_AlC_2_ powder was purchased from Beijing Lianlixin Technology Co., Ltd. Hydrochloric acid (HCl), lithium fluoride (LiF), potassium hydroxide (KOH), dimethyl sulfoxide (DMSO), silver nitrate (AgNO_3_), copper sulfate (CuSO_4_), sodium hydroxide (NaOH), L( +)-tartaric acid potassium Sodium salt (C_4_H_4_KNaO_6_·4H_2_O), methanal (HCHO) were pursued from Sinopharm Chemical Reagent Co., LTD. Deionized (DI) water was also used during the experimentation.

### Preparation of Ti_3_C_2_T_x_ MXene Suspension

Ti_3_C_2_T_x_ MXene suspension was obtained by a chemical etching method described in our previous work [44]. Typically, Ti_3_AlC_2_ (1 g) was mixed with the etching solution composed of 9 M HCl and LiF (1 g) followed by magnetic stirring for 72 h at 50 °C in an oil bath. Subsequently, the obtained mixture was washed several times by deionized (DI) water with centrifugation at 9,000 rpm for 10 min to reach a neutral pH. After being freeze-dried (–55 °C, 5 Pa) for 48 h, the obtained powders were dispersed in DMSO solution by ultrasonic treatment for 180 min. Followed by the low-speed centrifugal treatment at 3,500 rpm for 30 min to remove the unexfoliated large particles, the Ti_3_C_2_T_x_ MXene suspension with controlled solid content of 2 wt% was finally obtained for further use.

### Preparation of Kevlar Polyanion Solution

Aramid pulp (2 g) was stirred intensely in the solvent composed of DMSO (94 mL), KOH (2 g) and DI water (4 mL) for 6 h; afterward, dark red color of Kevlar polyanion solution was formed with the solid content of 2 wt%.

### Preparation of TPU/Kevlar Polyanion/Ti_3_C_2_T_x_ MXene mixed solution

TPU granule (3 g) was firstly dissolved in 12 mL DMSO at 80 °C for 6 h to form a viscous solution, and then different weight of Kevlar polyanion solutions (0.5, 1.0, 1.5, and 2.0 g) was injected into TPU solution under mechanical stirring at 80 °C for 10 min, respectively. The four concentration gradients solutions were turned into high fluid state with the continuously stirring. Subsequently, the fixed amount of Ti_3_C_2_T_x_ MXene solution (0.5 g, 2 wt%) was added into the aforementioned solutions for 30 min, respectively. After being centrifuged at 5,000 rpm for 10 min to remove the bubble generated during the stirring process, TPU/Kevlar polyanion/Ti_3_C_2_T_x_ MXene mixed solution was ultimately achieved.

### Assembly of TPU/ANF/Ti_3_C_2_T_x_ MXene (PAM) Foams

The TPU/Kevlar polyanion/Ti_3_C_2_T_x_ MXene mixed solution was encapsulated in dialysis bag (MD 25) followed by the freezing process in a refrigerator for 1 h. Then, the solidified block was placed in coagulation bath using DI water under magnetic stirring for 12 h, the water was changed every 3 h and synchronically used as proton donor for the fabrication of ANF from Kevlar polyanion to form a solid hybrid bulk. Finally, the TPU/ANF/Ti_3_C_2_T_x_ MXene (PAM) foams were obtained after being dried in air oven at 40 °C for 12 h to remove the rest of DI water. The calculation results show that the four Kevlar polyanion occupied the PAM foams with 0.07, 0.13, 0.20, 0.26 wt%, and the corresponding PAM foams were named as PAM7, PAM13, PAM20, PAM26, respectively. For comparison, pure TPU foam and the TPU/Ti_3_C_2_T_x_ MXene (PM) composite foams were also fabricated by the same way.

### Electroless Deposition of Copper Nanoparticles on PAM Foams

Typically, the obtained PAM13 foam was dipped in AgNO_3_ solution (1 wt%) for 10 min, followed by washing with DI water to remove the excessive AgNO_3_ solution. Then PAM13 foam with Ag seeds was immersed in electroless plating solution (1.5 g CuSO_4_·5H_2_O, 1.20 g NaOH, 2.8 g C_4_H_4_KNaO_6_·4H_2_O and 3 mL HCHO) for different time of 0.5, 1.0, 1.5 and 2.0 h, respectively. After that, the electroless Cu plating PAM13 foams were obtained by drying in vacuum oven at 60 °C for 6 h and denoted as PAM13-Cu_0.5_, PAM13-Cu_1.0_, PAM13-Cu_1.5_ and PAM13-Cu_2.0_, respectively.

### Characterization

Scanning electron microscope (SEM, Phenom XL, Netherlands) was used to observe the microstructures of composite foams. X-ray photoelectron spectrometer (XPS, ESCALAB 250Xi, UK) was performed to trace the state of chemical element. X-ray diffraction (XRD) patterns were obtained with multi-function X-ray diffractometer (SmartLab, Japan) at a scan rate of 20° min^−1^. Fourier transform infrared spectroscopy (FTIR) spectra were recorded with infrared spectrometer (Nicolet 380, USA) with wavenumber from 400 to 4000 cm^−1^. Viscosity of the solution was performed by rheometer (Kinexus ultra + , UK). Mechanical properties were performed by INSTRON 5943 under different operating rate. The specific density of the composite foams was obtained by using a densimeter (ME103E, Switzerland). The average electrical conductivity of the foam was tested by a four-probe technique-based instrument (RTS-8, China) with five measurements in different locations. The current signal was obtained from a desktop multimeter (B2901A, USA). Storage modulus were tested by Dynamic Thermomechanical Analyzer (DMA7100, Japan); EMI SE was investigated by vector network analyzer (E5063A, USA) in X band (8.2–12.4 GHz). The S_11_ and S_21_ composed of real and imaginary parts were recorded to calculate the SE_*A*_, SE_*R*_, SE_*T*_, *R*, *T* and *A* coefficients by these formulas [45]:

1 $${\text{SE}}_{A} = 10\log \left( {\frac{{1{-}\left| {S_{11} } \right|^{2} }}{{\left| {S_{21} } \right|^{2} }}} \right)$$

2 $${\text{SE}}_{R} = 10\log \left( {\frac{1}{{1{-}\left| {S_{11} } \right|^{2} }}} \right)$$

3 $${\text{SE}}_{T} = {\text{SE}}_{A} + {\text{SE}}_{R} + {\text{SE}}_{M}$$

4 $$R = \left| {S_{11} } \right|^{2}$$

5 $$T = \left| {S_{21} } \right|^{2}$$

6 $$A = 1 - R - T$$

## Results and Discussion

**Fig. 1:**
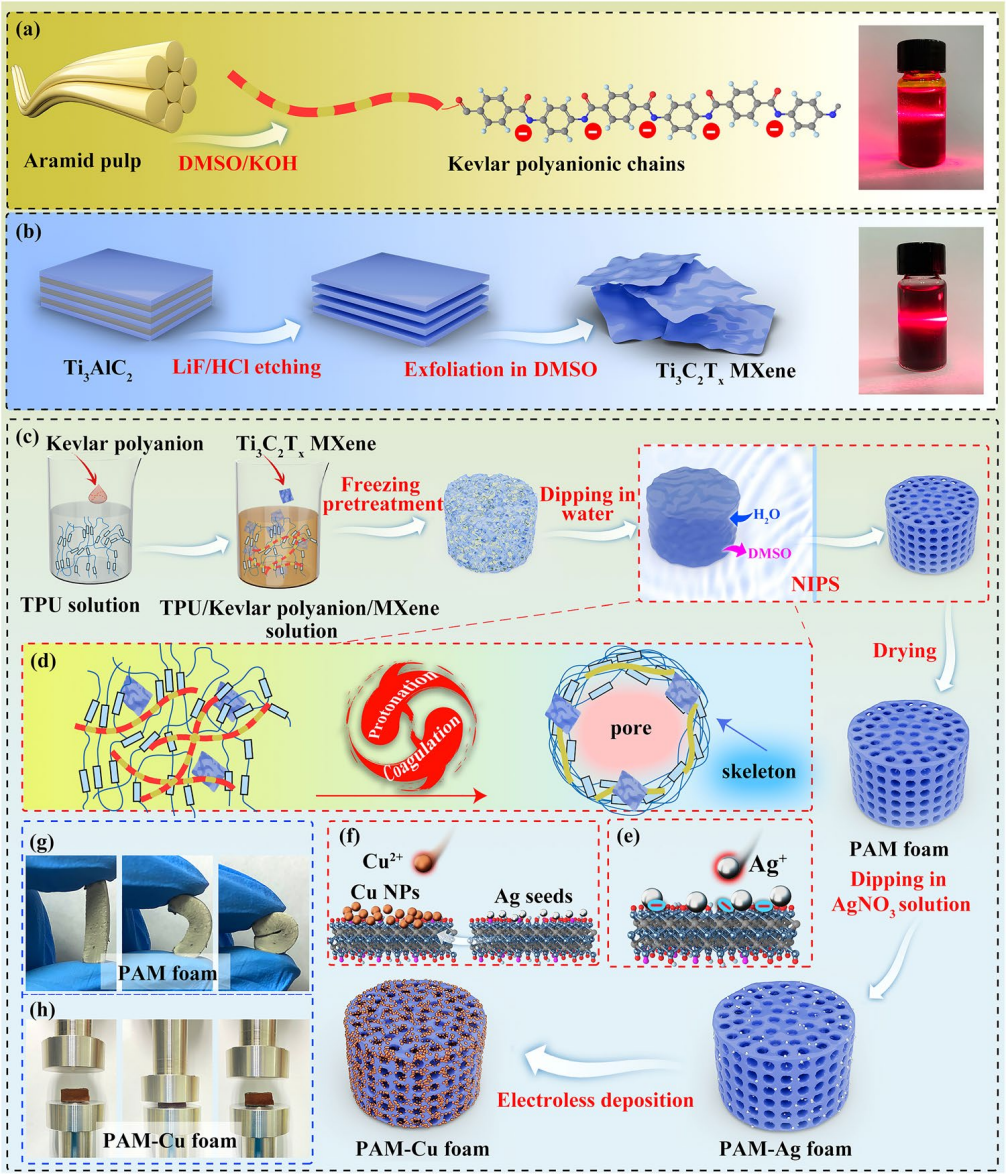
Illustrations of assembly process for highly ordered PAM-Cu conductive foam. Preparation of **a** Kevlar polyanion, **b** Ti_3_C_2_T_x_ MXene and **c** PAM-Cu foam. **d** Simultaneous process for protonation of Kevlar polyanion and coagulation of TPU. **e** Process for *in situ* reduction of Ag NPs on Ti_3_C_2_T_x_ MXene and **f** the electroless deposition of Cu NPs. **g** Digital pictures of PAM foam and **h** PAM-Cu foam

**Fig. 2:**
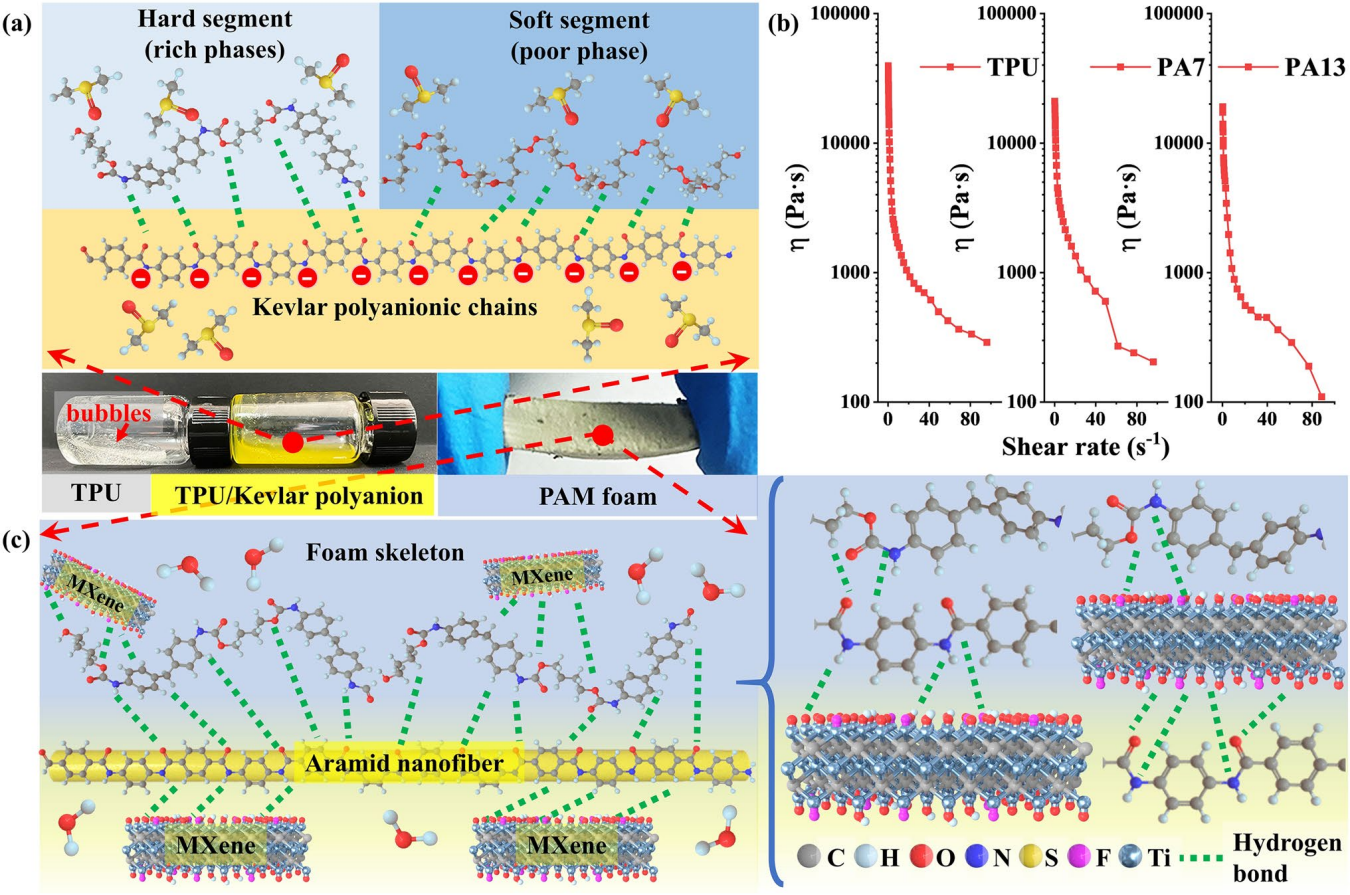
**a** Schematic of the bonding mechanism for TPU/Kevlar polyanionic chains. **b** Plots of viscosity versus shear rate of TPU, PA7 and PA13 solution. **c** Schematic of the bonding mechanism for PAM foam during NIPS process

Highly ordered PAM-Cu foams with homogeneously porous structure were fabricated by Kevlar polyanion modulated assembly strategy and the subsequent Ti_3_C_2_T_x_ MXene triggered electroless Cu deposition. Figure 1a is the typical top-to-bottom approach reported firstly by Yang et al. [46] for Kevlar polyanion obtained from the deprotonation and dissolution process of aramid pulp in DMSO/KOH solution . After the chemical cleavage of aramid fibers, the hydrogen from amide groups was extracted and then the hydrogen bonding interaction between polymer chains were accordingly reduced. Therefore, the electrostatic repulsion between polyanionic chains guaranteed the stability and homogeneity of Kevlar polyanion solution [47, 48]. Similarly, the exfoliated Ti_3_C_2_T_x_ MXene was obtained as expected (Fig. S1), displaying high dispersibility owing to the electrostatic repulsion caused by the negatively charged terminating groups on Ti_3_C_2_T_x_ MXene surface (Fig. 1b) [49]. The fabrication process for PAM-Cu foams is depicted in Fig. 1c. After being dissolved in DMSO, TPU solution presents high viscosity and poor fluidity with lots of bubbles as shown in the digital picture of Fig. 2, which can be ascribed to the high molecular weight and strong intermolecular force leading to the high internal friction resistance of polymer chains. Taking advantage of the isocyanate bonds (-NCO) in hard segment and polyol structure in soft segment of TPU, Kevlar polyanionic chains with deprotonated amido bonds could disperse uniformly by hydrogen bonding interaction (Fig. 2a). Interestingly, it is worth noting that the viscosity dramatically decreased after slight Kevlar polyanion introduced (Movie S1), and the viscosity plot further shows the dramatic decrease in viscosity after the addition of Kevlar polyanion in the whole range of shear rate (Fig. 2b). This finding may be attributed to the infiltration of Kevlar polyanionic chains in hard domains of TPU, resulting in a moderate molecular-to-lamellar-level ordering disruption, decreasing the interaction and flow resistance of TPU polymer chains; as a consequent, the decreased flow-activation energy efficiently upgraded the flexibility of TPU polymer chains [50]. Moreover, after the addition of Ti_3_C_2_T_x_ MXene, homogeneous TPU/Kevlar polyanion/Ti_3_C_2_T_x_ MXene solution was formed via hydrogen bonding interaction. Followed by the subsequent NIPS process, the structural collapse of the final foams without the freezing pretreatment (Fig. S2) was mainly due to the non-uniformity of infiltration distribution of DI water along thickness direction of the solution. Therefore, the freezing pretreatment was performed because crystalline DMSO formed in the refrigerator not only maintained bulk shape of the mixture but also promoted DI water infiltration during the melting process of the frozen block in DI water at room temperature. In the NIPS process, lots of water molecules infiltrated into TPU/Kevlar polyanion/Ti_3_C_2_T_x_ MXene mixture, facilitating the phase separation, namely, the rich phases eventually coagulated to form foam skeletons and the poor phases formed the porous structure [51, 52]. Simultaneously, the Kevlar polyanion could be easily transformed into ANF by hydrogen protons in DI water [53, 54] (Fig. 1d). Finally, PAM foams with high flexibility were obtained after being thoroughly dried as illustrated in Fig. 1g. When the PAM foams were immerged in AgNO_3_ solution, Ti_3_C_2_T_x_ MXene with the negatively charged groups subsequently anchored with Ag NPs by the van der Waals force [42, 55, 56] (Fig. 1e). The small amount of Ag NPs with the average size of 170 nm (Fig. S3) was served as the seeds (namely catalyst) for electroless Cu plating (Fig. 1f) to form electrically conductive networks and avoiding large usage of Ag noble metal. Consequently, conductive PAM-Cu foams with high compressibility were obtained (Fig. 1h).

**Fig. 3:**
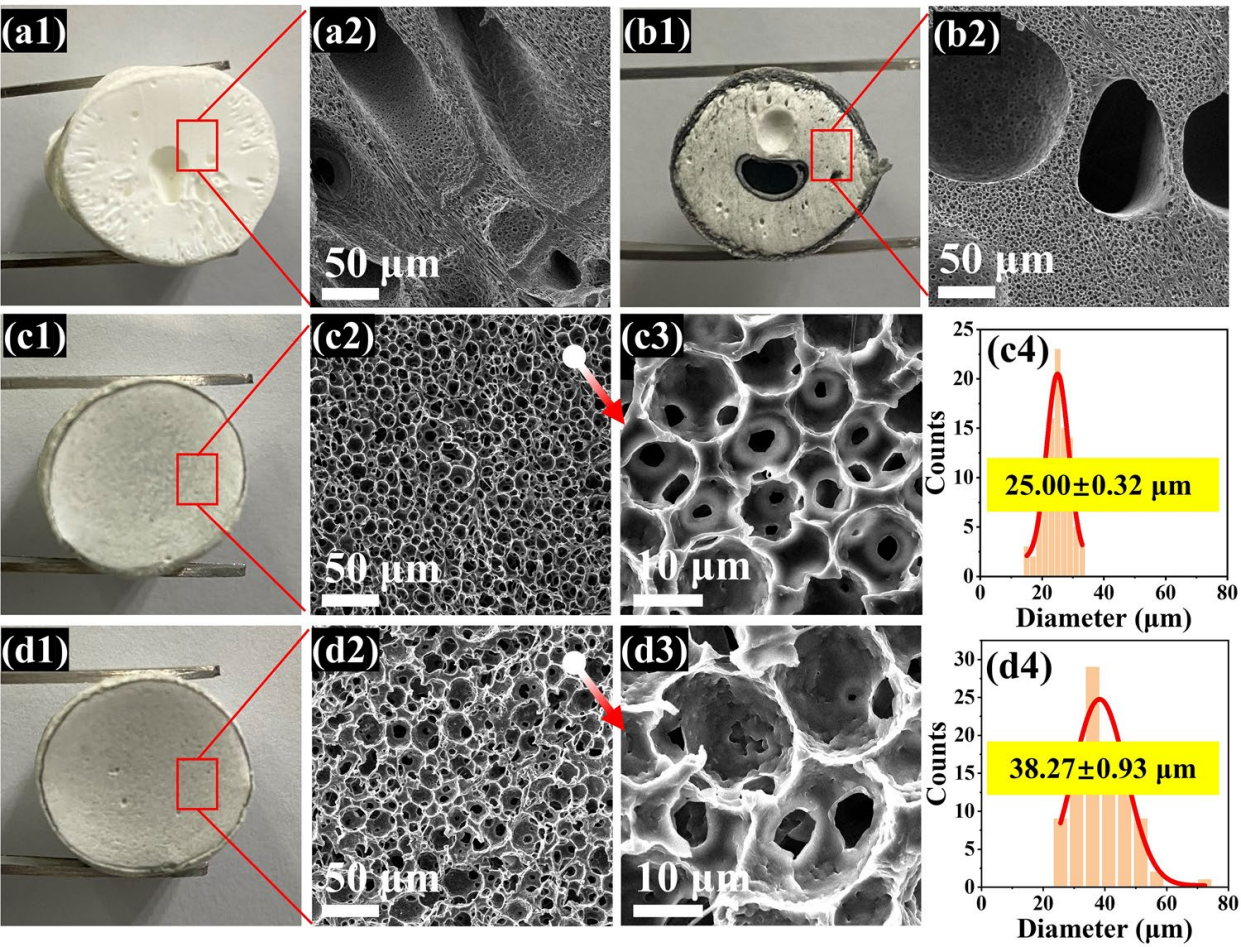
**a1-a2** Digital pictures and the corresponding SEM images of pure TPU foam. **b1-b2** Digital pictures and the corresponding SEM images of PM foam. **c1-c4** Digital pictures, the corresponding SEM images and pore size distribution of PAM7 foam. **d1-d4** Digital pictures, the corresponding SEM images and pore size distribution of PAM13 foam

During the protonation of Kevlar polyanion, ANF assembled quickly and aggregated as fibrous skeletons into the TPU matrix which facilitated the infiltration of water molecules effectively, thus avoiding the generation of macroporous structure caused by slow infiltration of water molecules. The successfully protonated ANF is about 50 nm in diameter (Fig. S4). Finally, Ti_3_C_2_T_x_ MXene was tightly attached into TPU skeletons by hydrogen bonding interaction as shown in Fig. 2c. Typical SEM images in Fig. 3 were used to investigate the morphological evolution of TPU-based foams. Both the pure TPU foam and PM foam displayed obviously macroporous structure visible to the naked eyes (Fig. 3a, b). The uniform dispersion of tiny amounts of Ti_3_C_2_T_x_ MXene was proved by elemental mapping images of Ti (Fig. S5). As shown in Fig. 3c, d, the optimized foam structure with homogeneous appearance and uniform pores is ascribed to the reduced viscosity of TPU solution modulated by Kevlar polyanion, so as to attenuate Marangoni convection to lower the thermodynamic gradient of hard domains and soft domains of TPU [24, 57]. Dramatically, with the introduction of different content of Kevlar polyanion, the size of pore was significantly improved from 25.00 ± 0.32 μm (PAM7) to 38.27 ± 0.93 μm (PAM13). It can be deduced from that the DI water in the protonation process promoted the process of phase separation; meanwhile, more Kevlar polyanion increased the heterogeneous nucleation sites, leading to the expansion of pore size during NIPS process. With the further increasing Kevlar polyanion contents, the over-cumulative ANF broken the affinity with TPU matrix, resulting in the structural collapse of PAM20 and PAM26 foams (Fig. S6).

**Fig. 4:**
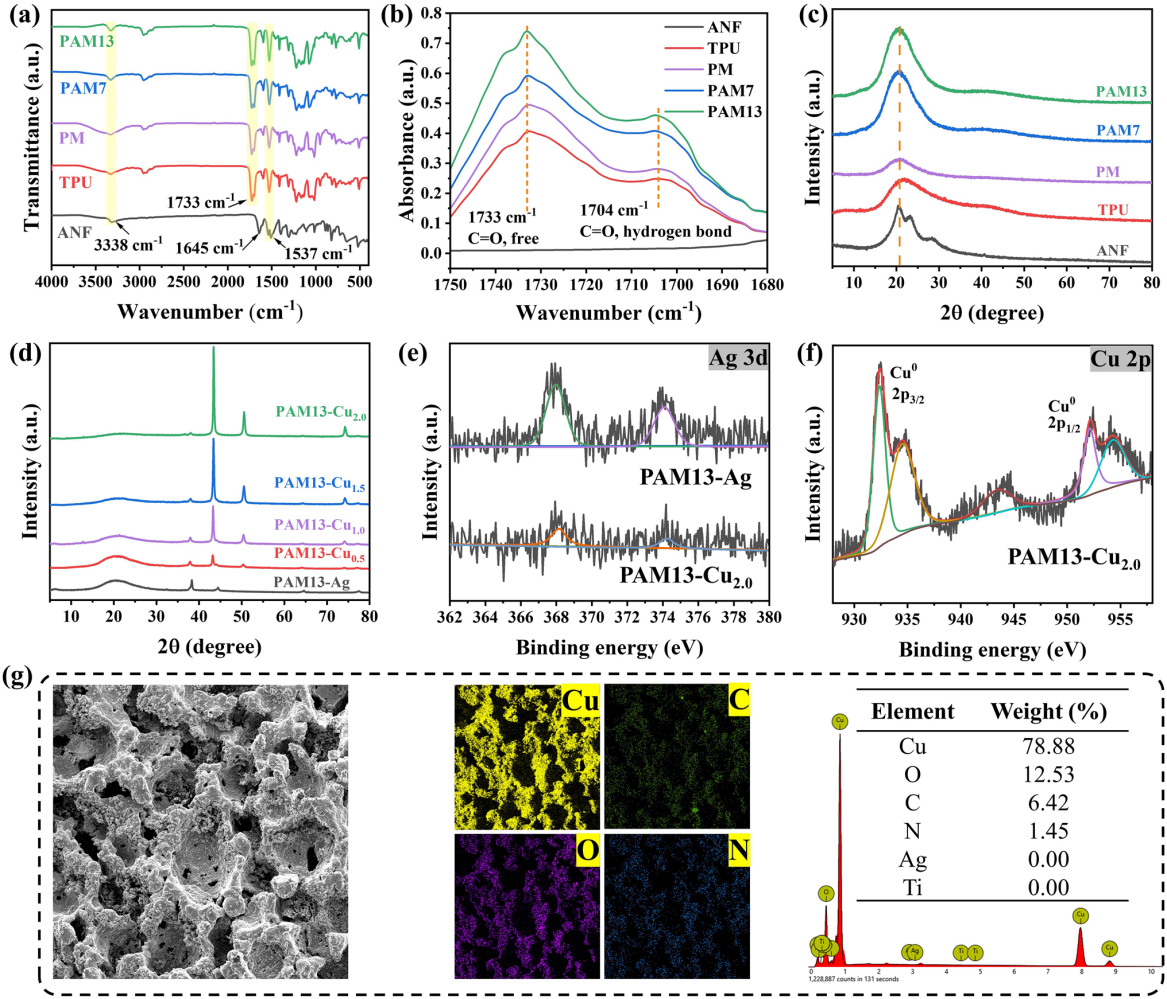
**a-b** IR spectra and **c** XRD patterns of ANF, TPU, PM, PAM7 and PAM13 foam. **d** XRD patterns of PAM13-Ag, PAM13-Cu_0.5_, PAM13-Cu_1.0_, PAM13-Cu_1.5_ and PAM13-Cu_2.0_ foam. **e** Ag 3d XPS spectra of PAM13-Ag and PAM13-Cu_2.0_ foams. **f** Cu 2p XPS spectrum of PAM13-Cu_2.0_ foam. **g** Element mapping and weight of PAM13-Cu2.0 foam

**Fig. 5:**
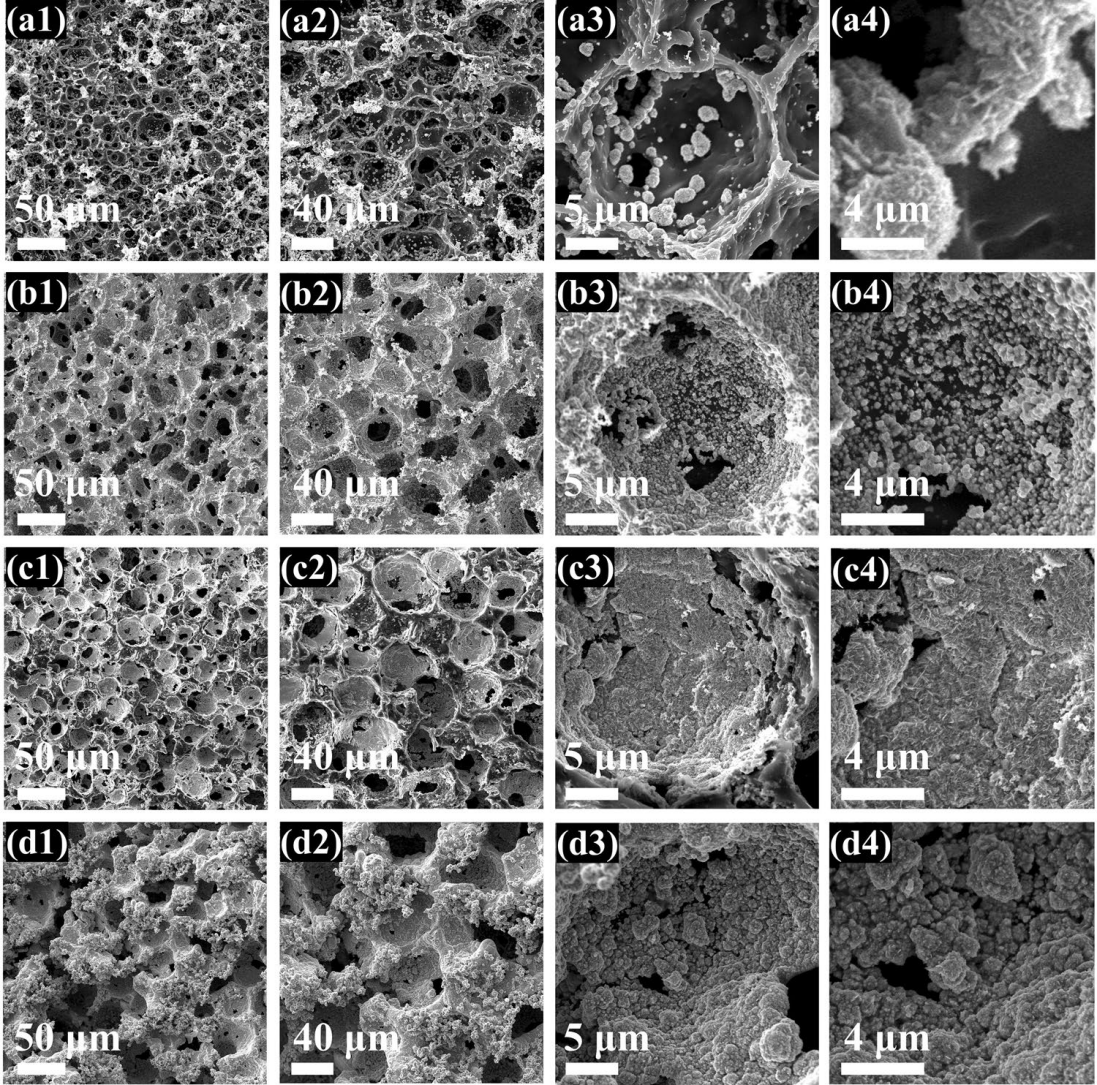
**a1-a4** SEM images of PAM13-Cu_0.5_ foam, **b1-b4** PAM13-Cu_1.0_ foam, **c1-c4** PAM13-Cu_1.5_ foam and **d1-d4** PAM13-Cu_2.0_ foam

In IR spectra (Fig. 4a), for all the samples, the typical peak at around 3338 and 1537 cm^−1^ can be assigned to N–H stretching and N–H bending vibrations. Though the similar C = O deformation of ANF (around 1645 cm^−1^) and TPU foam (around 1733 cm^−1^) is also observed, PM and PAM foam is hard to discern the existence of C = O of ANF due to the slight content of Kevlar polyanion. Specifically, free and hydrogen-bonded C = O appeared in 1733 and 1704 cm^−1^ in Fig. 4b is utilized to calculate hydrogen bonding index of C = O for the evaluation of microphase separation [58, 59]. According to the increased peak intensity at 1733 and 1704 cm^−1^ with the increasing of Kevlar polyanion, the as-obtained ANF promotes the generation of hydrogen bonds in the hard domains, which resulted in a significant phase separation between the hard and soft domains of TPU by the degree of phase separation (DSP) [60], as shown in Table S1. From XRD patterns (Fig. 4c), the peaks at 21.74° can be indexed to TPU, while 20.60°, 23.28° and 28.36° are attributed to aramid [61, 62]. Interestingly, the decreased peak intensity observed from PM foam demonstrated the reduced density difference between hard and soft segments [50], while PAM7 and PAM13 foams both presented a higher peak intensity in comparison with TPU and PM foams. The result manifested that the addition of Kevlar polyanion promoted the flexibility of TPU polymer chains, providing more hydrogen bonding interaction between TPU chains and ANF. Furthermore, Kevlar polyanion was also acted as nucleation sites to improve crystallization capacity of PAM foam during the NIPS process. Simultaneously, the peak position of PAM7 and PAM13 foam shifted to small angle comparing with TPU foam, indicating that the as-obtained ANF was inserted into the hard domains of TPU to increase the intermolecular spacing [50, 63, 64]. In Fig. 4d, besides the characteristics peak of TPU, the typical XRD peaks of PAM-Ag at 38.30°, 44.46°, 64.58° and 77.50° corresponding to (111), (200), (220) and (311) lattice planes of silver are also detected [65], confirming that Ag NPs were successfully anchored on PAM foams. With increasing electroless Cu plating time from 0.5 h to 2.0 h, the peaks at 43.28°, 50.52° and 74.18° corresponding to (111), (200) and (220) planes of face-centered cubic structure copper become stronger [66, 67]. The result can also be observed from Ag 3d XPS spectra of PAM13-Ag and PAM13-Cu_2.0_ foam, the peak of Ag 3d becomes lower after the deposition of Cu NPs (Fig. 4e). Moreover, XPS spectrum of Cu and the elements mapping further verify the successful deposition of interconnected Cu NPs layer on the surface of PAM13 foam (Fig. 4f, g). With the deposition time of 0.5 h, loosely distributed Cu NPs were observed on PAM13-Cu_0.5_ foam; however, an accumulated Cu NPs layer was accordingly coated on the surface of PAM13-Cu_2.0_ with the further increasing deposition time to 2 h (Fig. 5).

**Fig. 6:**
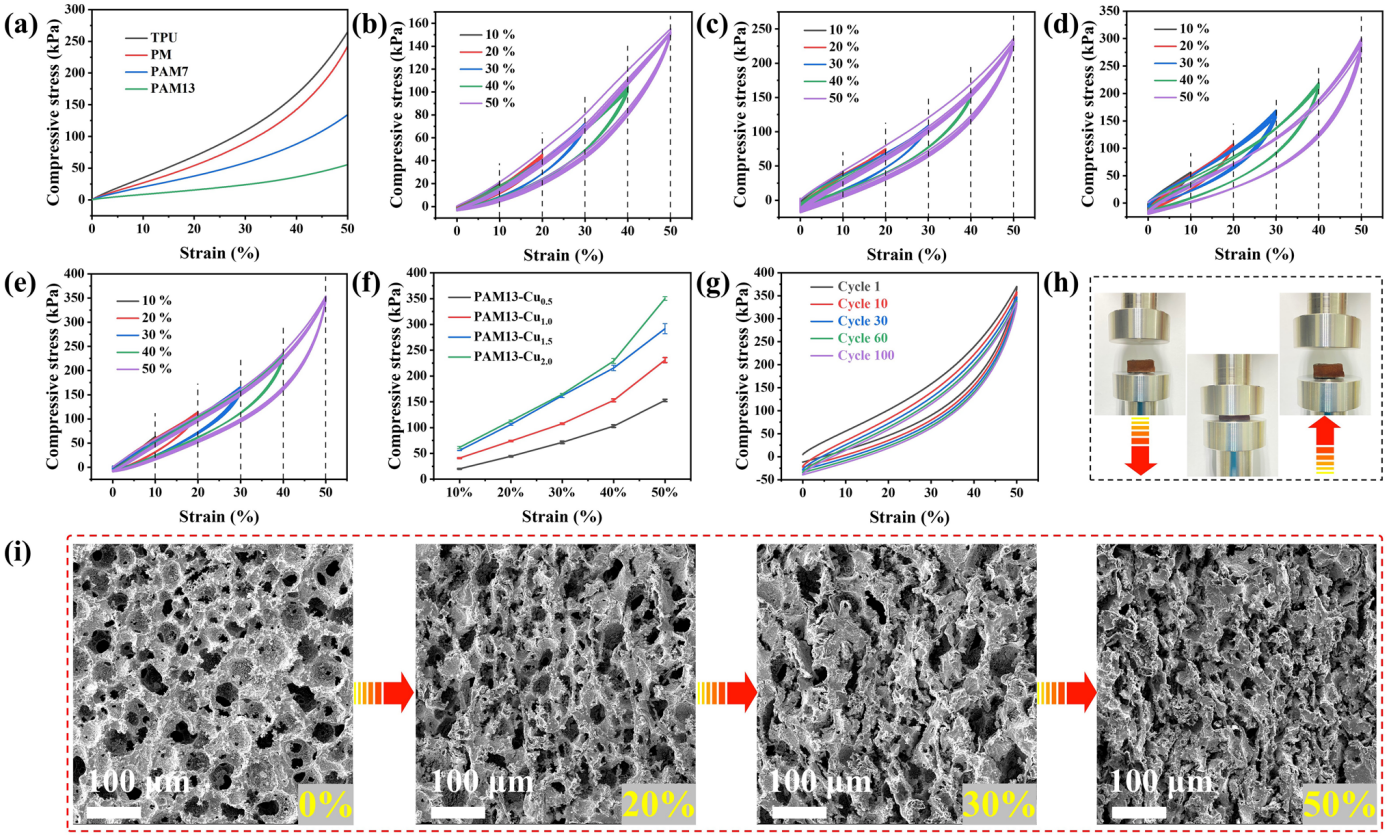
**a** Compressive stress–strains curve of TPU, PM, PAM7 and PAM13 foams. **b-e** Loading–unloading cycles of compressive stress–strain curves and **f** maximum compressive stress of PAM13-Cu_0.5_, PAM13-Cu_1.0_, PAM13-Cu_1.5_ and PAM13-Cu_2.0_ foams at 10%, 20%, 30%, 40% and 50% strain. **g** Loading–unloading cycles of compressive stress–strains curves of PAM13-Cu_2.0_ foam for 100 cycles. **h** Digital pictures of PAM13-Cu_2.0_ foam during the compressive process. **i** SEM images of PAM13-Cu_2.0_ foam with 0%, 20%, 30% and 50% strain

The compressive performance of the conductive porous foams is considered as a key role in practical application. So, the mechanical compressive ability of TPU, PM, PAM7 and PAM13 foams is given in Fig. 6a, and the relative stress–strain curves are divided into three stages [68]: The first liner stage (strain < 10%) is influenced by the elastic bending and accommodation of skeleton. With the increase in strain (10 < strain < 35%), a plateau region attributing to a slow increase along with the porous structure gradually collapses in the foam. At the last stages (strain > 35%), the compact internal structure endows the mechanical stress increasing sharply. Due to the biggest pore size structures, PAM13 displays the lowest stress within 50% strain. Particularly, the excellent cycling stability of PAM13-Cu foam upon loading–unloading cycles at different strain of 10, 20, 30, 40 and 50% was investigated. It can be found that all the PAM13-Cu_0.5_, PAM13-Cu_1.0_, PAM13-Cu_1.5_ and PAM13-Cu_2.0_ foams exhibited the promising structural stability after seven cycles and completely recovered to original state even after being compressed at the big strain (Fig. 6b-e). Specifically, the maximum compressive stress of PAM13-Cu foam at different strain reveals that the closely interconnected Cu NPs formed compact layer on the foam skeleton (Fig. 6f). Delightedly, after being compressed at the strain of 50% for 100 cycles, PAM13-Cu_2.0_ foam suffered negligible structure deformation (Fig. 6g), indicating the ultrahigh compressibility and good recoverability (Fig. 6h). The structure stability is verified by the dynamic SEM images (Fig. 6i), PAM13-Cu_2.0_ foam with different compressive strain still maintains the interconnected porous structure without collapse or shedding of Cu NPs, paving the way for potential application in piezoresistive sensors.

**Fig. 7:**
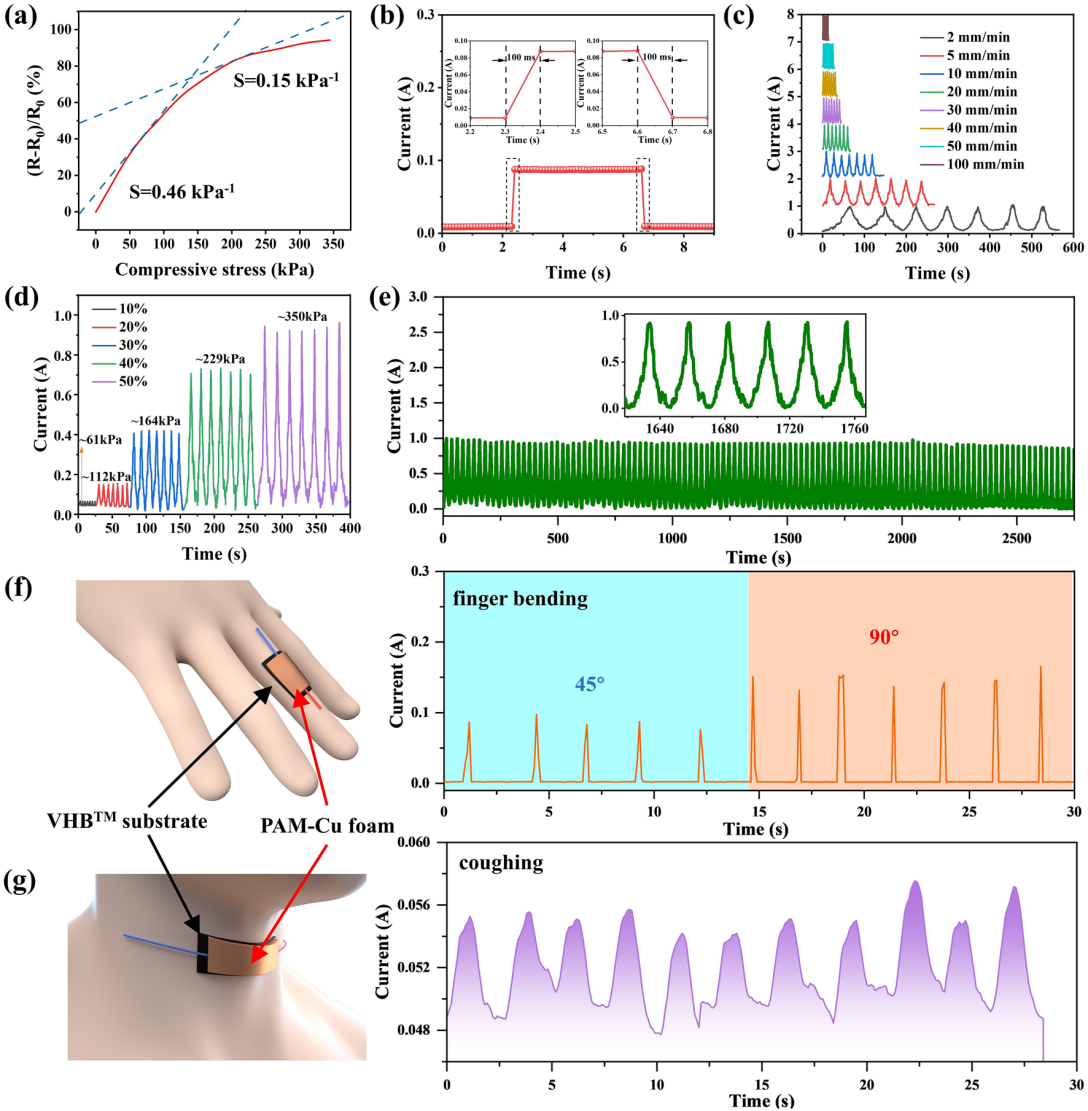
**a** Sensitivity of PAM13-Cu_2.0_ foam. **b** Response and recover time of PAM13-Cu_2.0_ foam upon applied with tiny touch. **c** Current signal of PAM13-Cu_2.0_ foam under different compressive rate and same compressive strain of 50%. **d** Current signal of PAM13-Cu_2.0_ foam under different compressive strain and same compressive rate of 10 mm min^−1^. **e** Cyclic stability performance of PAM13-Cu_2.0_ foam for 300 cycles (3,000 s). The current signal under real-time monitoring of PAM13-Cu_2.0_ foam on: **f** finger bending, **g** throat coughing

From the pressure sensitivity (*S*) curve of PAM13-Cu_2.0_ foam (Fig. 7a), the interface contact was improved by very little pressure in a low-pressure range, causing resistance changes to achieve higher sensitivity. While the compressive stress exceeds 147 kPa, *S* value reduced from 0.46 to 0.15 kPa^−1^, attributing to the internal densification of the foam to form denser conductive network accordingly [69]. Figure 7b displays the fast response/recovery time of 100 ms under a tiny strain, respectively, indicating excellent resilience and structural stability [70]. The cyclic response performance of PAM13-Cu_2.0_ foam is presented in Fig. 7c. With the same compressive strain, the variation of current signal is consistent with the rapid change of compressive rate, while the maximum value of current change is not related to the compressive rate, indicating the stable conductive path of the porous structure. In the same way, under the different compressive strain with same compressive rate, the current signal varies in width and intensity (Fig. 7d). It can be deduced that remarkably repeatable and stable signals of all the cyclic current curves originated from the well-constructed conductive porous architectures, further demonstrating that PAM13-Cu_2.0_ foam possessed the capability of rapidly identifying different levels of compressive stress. The reusability of PAM13-Cu_2.0_ foam for piezoresistive sensor was estimated by loading–unloading cyclic compression at 50% strain for 300 cycles under a rate of 10 mm min^−1^ (Fig. 7f). The stable and almost identical current signals during the process of loading and releasing indicated that the PAM13-Cu_2.0_ foam possessed remarkable mechanical stability and excellent current response repeatability. Benefiting from the piezoresistive sensing characteristics and outstanding mechanical properties, the PAM13-Cu_2.0_ foam can be served as the sensor to monitor human motions in real time.

For motion detection, current signal increased rapidly when the action performed, and subsequently recovered to the initial state following by the release of PAM13-Cu_2.0_ foam. Figure 7g visually displays a finger bending under angle of 45° and 90°, the large bending angle prompts the contact of conductive porous structure and further reduces the contact resistance so that the current signal is increased visibly. Figure 7h shows different current signals comparing with finger bending caused by the smaller deformation. The normal throat coughing can also be monitored in regular characteristic waves, indicating the sensing capability for small motion detection. To summarize, the conductive foam with porous structure possesses good potential in monitoring human activities such as the patient's rehabilitation and human–computer interaction.

**Fig. 8:**
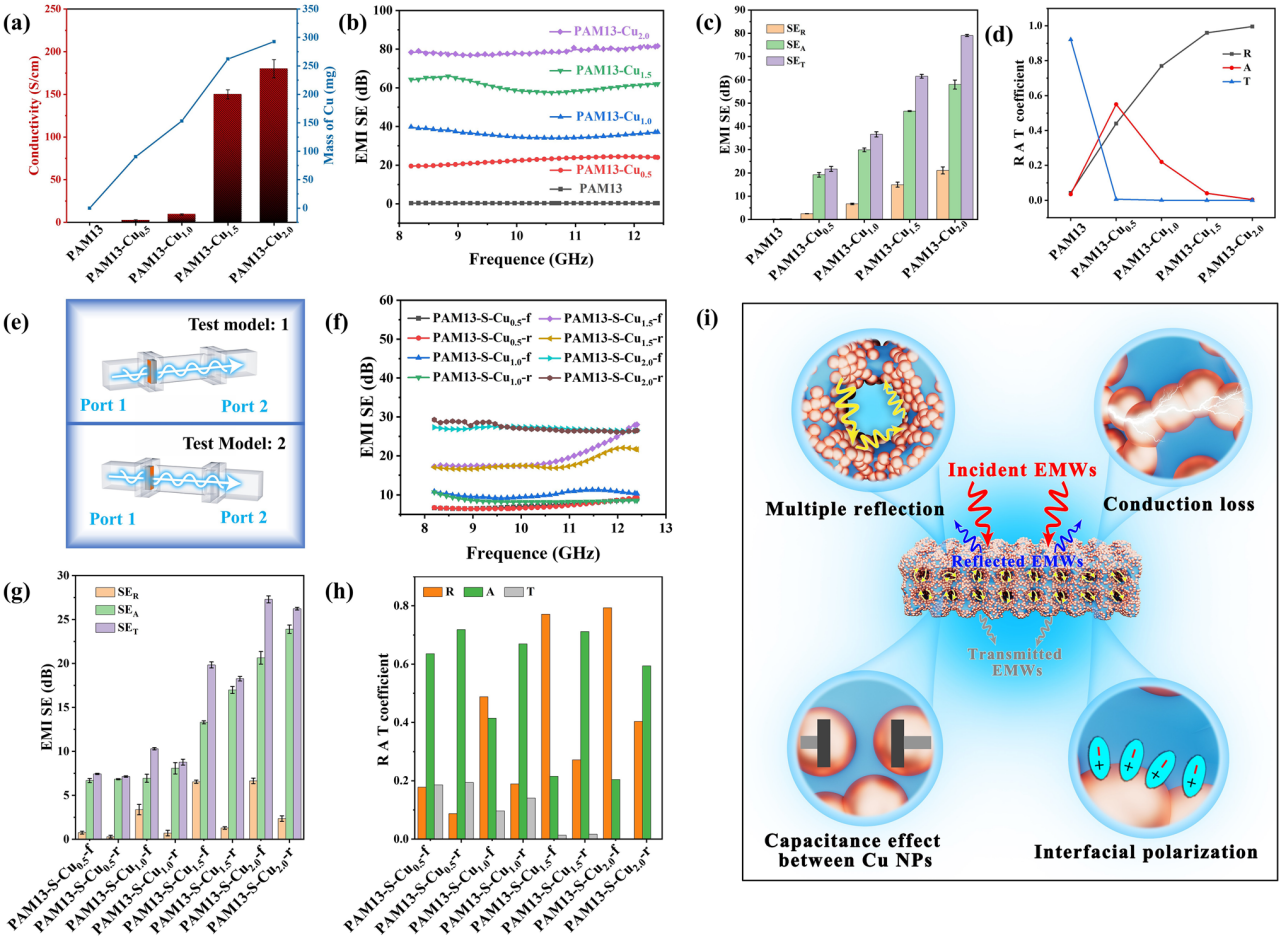
**a** Relationship between conductivity and deposited Cu content. **b** EMI SE of PAM, PAM13-Cu_0.5_, PAM13-Cu_1.0_, PAM13-Cu_1.5_ and PAM13-Cu_2.0_ foam. **c** Average SE_A_, SE_R_ and SE_T_ of PAM13-Cu_2.0_ foam. **d** Average *R*, *A* and *T* coefficients of PAM, PAM13-Cu_0.5_, PAM13-Cu_1.0_, PAM13-Cu_1.5_ and PAM13-Cu_2.0_ foam. **e** Test model for PAM13-Cu foam in vector network analyzer. **f** EMI SE, **g** average SE_A_, SE_R_ and SE_T_ and **h** average *R*, *A* and *T* coefficients of under different test models. **i** EMI shielding mechanism of PAM13-Cu foam

The orderly arranged porous structure PAM13-Cu foam composed of well-constructed conductive network is a promising candidate for high performance EMI shielding material. As shown in Fig. 8a, with the raising of ELD time, the conductivity of PAM13-Cu displays a rapid upward trend due to the increased mass of Cu NPs (details listed in Table S2). Simultaneously, the average EMI SE in X band of PAM13-Cu_0.5_, PAM13-Cu_1.0_, PAM13-Cu_1.5_ and PAM13-Cu_2.0_ foam reaches 21.74, 36.61, 61.54 and 79.09 dB, respectively (Fig. 8b), while the EMI SE of PAM13 foam without the deposition of Cu NPs is nearly 0 dB, indicating that the little content of Ti_3_C_2_T_x_ MXene has no contribution on EMI shielding. To explore the underlying EMI shielding mechanism of PAM13-Cu foams, the SE_*T*_, SE_*R*_ and SE_*A*_ values were calculated, respectively (Fig. 8c), and the typical coefficients of reflection (*R*), absorption (*A*), and transmission (*T*) were also analyzed individually (Fig. 8d). After the deposition of Cu NPs, *R* is always higher than *A* for all the PAM13-Cu foams, indicating that PAM13-Cu foams reflected most of incident EMWs by the impedance mismatch with air and conduction loss. Furthermore, to effectively investigate the effect of porous structure in PAM13-Cu foams, PAM13 foams were deposited with Cu NPs at only single face for 0.5, 1.0, 1.5, 2.0 h, respectively. As such, two models were proposed as shown in Fig. 8e: The first is the deposited single face as the front face to the port 1 of vector network analyzer (named as PAM13-S-Cu-f), and the second is the deposited single face as the reverse face to the port 1 of vector network analyzer (named as PAM13-S-Cu-r). With the same deposition time, the average EMI SE of PAM13-S-Cu-f foam is slightly higher than that of PAM13-S-Cu-r foam (Fig. 8f), and specifically, the tiny SE_T_ advantage of PAM13-S-Cu-f foam owes to the high SE_R_ (Fig. 8g), which is also revealed by the higher *R* displayed in Fig. 8h. For PAM13-S-Cu-f foam, EMWs were directly reflected by the high impedance mismatch between air and surface conductive layer. But PAM13-S-Cu-r foam with nearly similar EMI SE value is attributed to the higher SE_*A*_ and *A*. The EMWs across to the whole porous structures were reflected by the impedance mismatch between the insulating part and the deposited conductive layer. Moreover, the reflected EMWs were dissipated by the inner porous structure via the interfacial polarization. Moreover, the EMI shielding stability of PAM13-Cu foam after loading–unloading cyclic compression at 50% strain for 300 cycles was investigated, and it can be found that the average EMI SE dropped slightly from 79.09 to 73.39 dB (Fig. S7), indicating the excellent mechanical durability of the conductive foams. The remarkable EMI shielding performance of the PAM13-Cu foam is absolutely relied on the multiple structure from macroscale to microscale (Fig. 8i). On the macroscale, the incident EMWs were reflected immediately by the impedance mismatch, and then the remaining EMWs entered the inner of the foams and significantly attenuated by the repeated reflections between the top and bottom conductive layers. On the microscale, large amounts of microscale conductive porous structure extended the propagation path of EMWs via the internal multiple reflections [71]. The EMWs interacted with high density of electron carriers in the surface of Cu layers, leading to the excellent conduction loss; therefore, the EMWs were attenuated by induced currents and dissipated as heat at a great extent [72, 73]. Furthermore, in the magnetic field, interfacial polarization was induced and caused charge accumulation between the heterogeneous interface of Cu layers and insulating parts, to significantly increase the absorption of EMWs. On the nanometer scale, an aggregation of large numbers of Cu NPs on the surface of porous skeleton formed abundant microcapacitances to consume EMWs efficiently [74, 75]. Besides, surface defects of Cu NPs on the porous surface also led to the attenuation of EMWs. In addition, owing to the surface plasmon resonance of Cu NPs, that is, the frequency of the incident EMWs is close to the plasmon resonance frequency of metallic nanoparticles, the EMWs were absorbed accordingly [76].

**Fig. 9:**
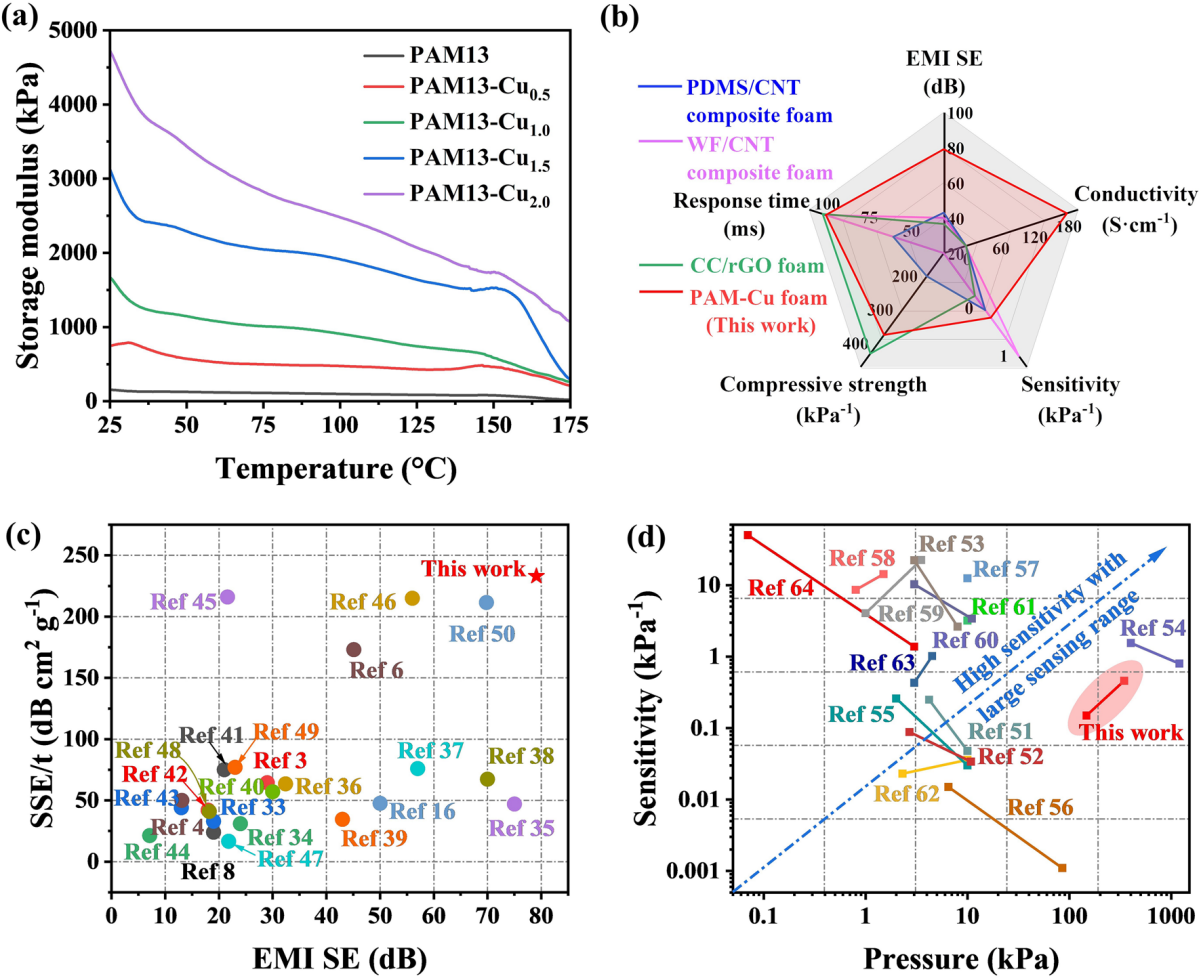
**a** Plots of storage modulus versus temperature. **b** Radar chart of EMI shielding and piezoelectric sensor performance among common composite foam. **c** Comparison of EMI shielding performance of various composite foams ever reported (the reference number listed in Supporting Information). **d** Reported piezoelectric sensor with pressure and sensitivity (the reference number listed in Supporting Information)

The dynamic mechanical properties of PAM13-Cu foams were further investigated as shown in Fig. 9a. Compared with PAM foam, PAM13-Cu foam exhibited outstanding higher storage modulus (387, 954, 1,887 and 2,932% increment than PAM, respectively). Results demonstrated that the introduction of Cu NPs as protective armor increased the surface roughness of PAM pore and meanwhile hindered the movement of polymer chains. With the further increase in Cu NPs content, the interface binding effect became more obvious, resulting in a higher storage modulus, which is conducive to various promising applications under high compressive conditions [77]. The comprehensive performance of the multi-functional foams possessed EMI shielding and piezoresistive sensor are compared in Fig. 9b [78–80]. It is obvious that PAM-Cu foams hold good promise as potentials in EMI shielding with high compressive strength and storage modulus. By comparing with other composite materials, the highly ordered porous PAM-Cu foams with excellent conductivity exhibited the predominant advantages combined with remarkable sensitivity and noteworthy EMI shielding performances, particularly in large compressive deformation applications (Fig. 9c, d).

## Conclusions

Highly ordered PAM-Cu foams with adjustable pore size were successfully fabricated by using Kevlar polyanionic chains induced assembly via a simple NIPS process, followed by the coating of Cu NPs layers according to the *in situ* reduction of Cu NPs using by tiny amounts of Ti_3_C_2_T_x_ MXene as reducing agent. Benefitting from the high elasticity of TPU and the orderly microscale pore structure, the PAM-Cu foams showed highly promising potential properties in detecting human motions, including board compressive interval of 0–344.5 kPa (50% strain) with good sensitivity at 0.46 kPa^−1^, and rapid response/recovery time of 100 ms, respectively. Meanwhile, the PAM-Cu foams manifested remarkable EMI SE at 79.09 dB in X band, displaying outstanding absorption capacity of EMWs due to the well-constructed porous conductive structure. The well-designed PAM-Cu foams in our work with the integration of performances in piezoresistive sensing and EMI shielding are expected to facilitate the development of human–machine interfaces, artificial intelligence, flexible wearable electronic devices and other high-tech fields.

### Supplementary Information

Below is the link to the electronic supplementary material.

Supplementary file1 (PDF 982 kb)

Supplementary file2 (MP4 7266 kb)

## References

[1] Luo W, Wang M, Wang K, Yan P, Huang J (2022). A robust hierarchical MXene/Ni/aluminosilicate glass composite for high-performance microwave absorption. Adv. Sci..

[2] Zeng Z, Wu N, Yang W, Xu H, Liao Y (2022). Sustainable-macromolecule-assisted preparation of cross-linked, ultralight, flexible graphene aerogel sensors toward low-frequency strain/pressure to high-frequency vibration sensing. Small.

[3] Zhou J, Thaiboonrod S, Fang J, Cao S, Miao M (2022). In-situ growth of polypyrrole on aramid nanofibers for electromagnetic interference shielding films with high stability. Nano Res..

[4] Wang L, Shi X, Zhang J, Zhang Y, Gu J (2020). Lightweight and robust rGO/sugarcane derived hybrid carbon foams with outstanding EMI shielding performance. J. Mater. Sci. Technol..

[5] Kuang T, Ju J, Chen F, Liu X, Zhang S (2022). Coupled effect of self-assembled nucleating agent, Ni-CNTs and pressure-driven flow on the electrical, electromagnetic interference shielding and thermal conductive properties of poly (lactic acid) composite foams. Compos. Sci. Technol..

[6] Hou T, Jia Z, Dong Y, Liu X, Wu G (2022). Layered 3D structure derived from MXene/magnetic carbon nanotubes for ultra-broadband electromagnetic wave absorption. Chem. Eng. J..

[7] Zhang Y, Gu J (2022). A perspective for developing polymer-based electromagnetic interference shielding composites. Nano-Micro Lett..

[8] Gao J, Ding Q, Yan P, Liu Y, Huang J (2022). Highly improved microwave absorbing and mechanical properties in cold sintered ZnO by incorporating graphene oxide. J. Eur. Ceram. Soc..

[9] Kang KS, Phan A, Olikagu C, Lee T, Loy DA (2021). Segmented polyurethanes and thermoplastic elastomers from elemental sulfur with enhanced thermomechanical properties and flame retardancy. Angew. Chem. Int. Ed..

[10] Xiao M, Yao Y, Fan C, Xu Z, Liu Y (2021). Multiple H-bonding chain extender-based polyurethane: Ultrastiffness, hot-melt adhesion, and 3D printing finger orthosis. Chem. Eng. J..

[11] Hossieny NJ, Barzegari MR, Nofar M, Mahmood SH, Park CB (2014). Crystallization of hard segment domains with the presence of butane for microcellular thermoplastic polyurethane foams. Polymer.

[12] Nema AK, Deshmukh AV, Palanivelu K, Sharma SK, Malik T (2008). Effect of exo- and endothermic blowing and wetting agents on morphology, density and hardness of thermoplastic polyurethanes foams. J. Cell. Plast..

[13] Ni G-L, Zhu X, Mi H-Y, Feng P-Y, Li J (2021). Skinless porous films generated by supercritical CO_2_ foaming for high-performance complementary shaped triboelectric nanogenerators and self-powered sensors. Nano Energy.

[14] Jun Y-S, Hyun BG, Hamidinejad M, Habibpour S, Yu A (2021). Maintaining electrical conductivity of microcellular MWCNT/TPU composites after deformation. Composites Part B.

[15] Fei Y, Chen F, Fang W, Xu L, Ruan S (2020). High-strength, flexible and cycling-stable piezo-resistive polymeric foams derived from thermoplastic polyurethane and multi-wall carbon nanotubes. Composites Part B.

[16] Lei Z, Tian D, Liu X, Wei J, Rajavel K (2021). Electrically conductive gradient structure design of thermoplastic polyurethane composite foams for efficient electromagnetic interference shielding and ultra-low microwave reflectivity. Chem. Eng. J..

[17] Sang G, Xu P, Yan T, Murugadoss V, Naik N (2021). Interface engineered microcellular magnetic conductive polyurethane nanocomposite foams for electromagnetic interference shielding. Nano-Micro Lett..

[18] Li Y, Pei X, Shen B, Zhai W, Zhang L (2015). Polyimide/graphene composite foam sheets with ultrahigh thermostability for electromagnetic interference shielding. RSC Adv..

[19] Shen B, Zhai W, Tao M, Ling J, Zheng W (2013). Lightweight, multifunctional polyetherimide/graphene@Fe_3_O_4_ composite foams for shielding of electromagnetic pollution. ACS Appl. Mater. Interfaces.

[20] Zhao J, Luo G, Wu J, Xia H (2013). Preparation of microporous silicone rubber membrane with tunable pore size via solvent evaporation-induced phase separation. ACS Appl. Mater. Interfaces.

[21] Tree DR, Iwama T, Delaney KT, Lee J, Fredrickson GH (2018). Marangoni flows during nonsolvent induced phase separation. ACS Macro Lett..

[22] Qian HL, Huang WP, Fang Y, Zou LY, Yu WJ (2021). Fabrication of “spongy skin” on diversified materials based on surface swelling non-solvent-induced phase separation. ACS Appl. Mater. Interfaces.

[23] Garcia JU, Iwama T, Chan EY, Tree DR, Delaney KT (2020). Mechanisms of asymmetric membrane formation in nonsolvent-induced phase separation. ACS Macro Lett..

[24] Muller M, Abetz V (2021). Nonequilibrium processes in polymer membrane formation: Theory and experiment. Chem. Rev..

[25] Zhang C, Lv Q, Liu Y, Wang C, Wang Q (2021). Rational design and fabrication of lightweight porous polyimide composites containing polyaniline modified graphene oxide and multiwalled carbon nanotube hybrid fillers for heat-resistant electromagnetic interference shielding. Polymer.

[26] Han Y, Ruan K, Gu J (2022). Janus (BNNS/ANF)-(AgNWs/ANF) thermal conductivity composite films with superior electromagnetic interference shielding and Joule heating performances. Nano Res..

[27] Xu L, Zhao X, Xu C, Kotov NA (2018). Water-rich biomimetic composites with abiotic self-organizing nanofiber network. Adv. Mater..

[28] Guan Y, Li W, Zhang Y, Shi Z, Tan J (2017). Aramid nanofibers and poly (vinyl alcohol) nanocomposites for ideal combination of strength and toughness via hydrogen bonding interactions. Compos. Sci. Technol..

[29] Kuang Q, Zhang D, Yu JC, Chang Y-W, Yue M (2015). Toward record-high stiffness in polyurethane nanocomposites using aramid nanofibers. J. Phys. Chem. C.

[30] Wang H, Zhou R, Li D, Zhang L, Ren G (2021). High-performance foam-shaped strain sensor based on carbon nanotubes and Ti_3_C_2_T_x_ MXene for the monitoring of human activities. ACS Nano.

[31] Zeng ZH, Wu N, Wei JJ, Yang YF, Wu TT (2022). Porous and ultra-flexible crosslinked MXene/polyimide composites for multifunctional electromagnetic interference shielding. Nano-Micro Lett..

[32] Ma Z, Kang S, Ma J, Shao L, Zhang Y (2020). Ultraflexible and mechanically strong double-layered aramid nanofiber-Ti_3_C_2_T_x_ MXene/silver nanowire nanocomposite papers for high-performance electromagnetic interference shielding. ACS Nano.

[33] Zeng Z, Wu T, Han D, Ren Q, Siqueira G (2020). Ultralight, flexible, and biomimetic nanocellulose/silver nanowire aerogels for electromagnetic interference shielding. ACS Nano.

[34] Shahzad F, Alhabeb M, Hatter CB, Anasori B, Man HS (2016). Electromagnetic interference shielding with 2D transition metal carbides (MXenes). Science.

[35] Sun C, Jia Z, Xu S, Hu D, Zhang C (2022). Synergistic regulation of dielectric-magnetic dual-loss and triple heterointerface polarization via magnetic MXene for high-performance electromagnetic wave absorption. J. Mater. Sci. Technol..

[36] Qian K, Li S, Fang J, Yang Y, Cao S (2022). C_60_ intercalating Ti_3_C_2_T_x_ MXenes assisted by γ-cyclodextrin for electromagnetic interference shielding films with high stability. J. Mater. Sci. Technol..

[37] Liu L, Guo R, Gao J, Ding Q, Fan Y (2022). Mechanically and environmentally robust composite nanofibers with embedded MXene for wearable shielding of electromagnetic wave. Compos. Commun..

[38] Wang Z-X, Han X-S, Zhou Z-J, Meng W-Y, Han X-W (2021). Lightweight and elastic wood-derived composites for pressure sensing and electromagnetic interference shielding. Compos. Sci. Technol..

[39] Weng C, Wang G, Dai Z, Pei Y, Liu L (2019). Buckled AgNW/MXene hybrid hierarchical sponges for high-performance electromagnetic interference shielding. Nanoscale.

[40] Wan Y-J, Rajavel K, Li X-M, Wang X-Y, Liao S-Y (2021). Electromagnetic interference shielding of Ti_3_C_2_T_x_ MXene modified by ionic liquid for high chemical stability and excellent mechanical strength. Chem. Eng. J..

[41] Xiang Z, Shi Y, Zhu X, Cai L, Lu WJN-ML (2021). Flexible and waterproof 2D/1D/0D construction of MXene-based nanocomposites for electromagnetic wave absorption, EMI shielding, and photothermal conversion. Nano-Micro Lett..

[42] Satheeshkumar E, Makaryan T, Melikyan A, Minassian H, Gogotsi Y (2016). One-step solution processing of Ag, Au and Pd@MXene hybrids for SERS. Sci. Rep..

[43] Lan L, Jiang C, Yao Y, Ping J, Ying Y (2021). A stretchable and conductive fiber for multifunctional sensing and energy harvesting. Nano Energy.

[44] Qian K, Zhou Q, Wu H, Fang J, Miao M (2021). Carbonized cellulose microsphere@void@MXene composite films with egg-box structure for electromagnetic interference shielding. Compos. Part A.

[45] Wan Y-J, Zhu P-L, Yu S-H, Sun R, Wong C-P (2017). Graphene paper for exceptional EMI shielding performance using large-sized graphene oxide sheets and doping strategy. Carbon.

[46] Yang M, Cao K, Sui L, Qi Y, Zhu J (2011). Dispersions of aramid nanofibers: a new nanoscale building block. ACS Nano.

[47] Yang B, Li W, Zhang M, Wang L, Ding X (2021). Recycling of high-value-added aramid nanofibers from waste aramid resources via a feasible and cost-effective approach. ACS Nano.

[48] Zhang Y, Ruan K, Zhou K, Gu J (2023). Controlled distributed Ti_3_C_2_T_x_ hollow microspheres on thermally conductive polyimide composite films for excellent electromagnetic interference shielding. Adv. Mater..

[49] Zhang Y, Ruan K, Gu J (2021). Flexible sandwich-structured electromagnetic interference shielding nanocomposite films with excellent thermal conductivities. Small.

[50] Koo JM, Kim H, Lee M, Park S-A, Jeon H (2019). Nonstop monomer-to-aramid nanofiber synthesis with remarkable reinforcement ability. Macromolecules.

[51] Shin B, Mondal S, Lee M, Kim S, Huh Y-I (2021). Flexible thermoplastic polyurethane-carbon nanotube composites for electromagnetic interference shielding and thermal management. Chem. Eng. J..

[52] Okada I, Shiratori S (2014). High-transparency, self-standable gel-SLIPS fabricated by a facile nanoscale phase separation. ACS Appl. Mater. Interfaces.

[53] Yang B, Wang L, Zhang M, Luo J, Ding X (2019). Timesaving, high-efficiency approaches to fabricate aramid nanofibers. ACS Nano.

[54] Yang B, Wang L, Zhang M, Luo J, Lu Z (2020). Fabrication, applications, and prospects of aramid nanofiber. Adv. Funct. Mater..

[55] Schultz T, Frey NC, Hantanasirisakul K, Park S, May SJ (2019). Surface termination dependent work function and electronic properties of Ti_3_C_2_T_x_ MXene. Chem. Mater..

[56] Yao Y, Lan L, Liu X, Ying Y, Ping J (2020). Spontaneous growth and regulation of noble metal nanoparticles on flexible biomimetic MXene paper for bioelectronics. Biosens. Bioelectron..

[57] Rarima R, Unnikrishnan G (2020). Poly(lactic acid)/gelatin foams by non-solvent induced phase separation for biomedical applications. Polym. Degrad. Stab..

[58] Seymour R, Estes G, Cooper SLJM (1970). Infrared studies of segmented polyurethan elastomers. I. Hydrogen bonding. Macromol..

[59] Amin KNM, Chaleat C, Edwards G, Martin DJ, Annamalai PK (2022). A cleaner processing approach for cellulose reinforced thermoplastic polyurethane nanocomposites. Polym. Eng. Sci..

[60] Dong M, Li Q, Liu H, Liu C, Wujcik EK (2018). Thermoplastic polyurethane-carbon black nanocomposite coating: Fabrication and solid particle erosion resistance. Polymer.

[61] Yang Y, Lyu J, Chen J, Liao J, Zhang X (2021). Flame-retardant host–guest films for efficient thermal management of cryogenic devices. Adv. Funct. Mater..

[62] Zhang Y, Ma Z, Ruan K, Gu J (2022). Flexible Ti3C2Tx/(aramid nanofiber/PVA) composite films for superior electromagnetic interference shielding. Research..

[63] Liff SM, Kumar N, McKinley GH (2007). High-performance elastomeric nanocomposites via solvent-exchange processing. Nat. Mater..

[64] Stribeck A, Pöselt E, Eling B, Jokari-Sheshdeh F, Hoell A (2017). Thermoplastic polyurethanes with varying hard-segment components. Mechanical performance and a filler-crosslink conversion of hard domains as monitored by SAXS. Eur. Polym. J..

[65] Liang C, Liu Y, Ruan Y, Qiu H, Song P (2020). Multifunctional sponges with flexible motion sensing and outstanding thermal insulation for superior electromagnetic interference shielding. Composites Part A.

[66] Li J, Wang A, Qin J, Zhang H, Ma Z (2021). Lightweight polymethacrylimide@copper/nickel composite foams for electromagnetic shielding and monopole antennas. Composites Part A.

[67] Lu L, Wang B, Wu D, Zou S, Fang B (2021). Engineering porous Pd-Cu nanocrystals with tailored three-dimensional catalytic facets for highly efficient formic acid oxidation. Nanoscale.

[68] Zhang S, Liu H, Yang S, Shi X, Zhang D (2019). Ultrasensitive and highly compressible piezoresistive sensor based on polyurethane sponge coated with a cracked cellulose nanofibril/silver nanowire layer. ACS Appl. Mater. Interfaces.

[69] Ma Z, Xiang X, Shao L, Zhang Y, Gu J (2022). Multifunctional wearable silver nanowire decorated leather nanocomposites for joule heating, electromagnetic interference shielding and piezoresistive sensing. Angew. Chem. Int. Ed..

[70] Pu L, Liu Y, Li L, Zhang C, Ma P (2021). Polyimide nanofiber-reinforced Ti_3_C_2_T_x_ aerogel with “lamella-pillar” microporosity for high-performance piezoresistive strain sensing and electromagnetic wave absorption. ACS Appl. Mater. Interfaces.

[71] Ju J, Kuang T, Ke X, Zeng M, Chen Z (2020). Lightweight multifunctional polypropylene/carbon nanotubes/carbon black nanocomposite foams with segregated structure, ultralow percolation threshold and enhanced electromagnetic interference shielding performance. Compos. Sci. Technol..

[72] Luo J-Q, Zhao S, Zhang H-B, Deng Z, Li L (2019). Flexible, stretchable and electrically conductive MXene/natural rubber nanocomposite films for efficient electromagnetic interference shielding. Compos. Sci. Technol..

[73] Liang C, He J, Zhang Y, Zhang W, Liu C (2022). MOF-derived CoNi@C-silver nanowires/cellulose nanofiber composite papers with excellent thermal management capability for outstanding electromagnetic interference shielding. Compos. Sci. Technol..

[74] Gao Y-N, Wang Y, Yue T-N, Zhao B, Che R (2022). Superstructure silver micro-tube composites for ultrahigh electromagnetic wave shielding. Chem. Eng. J..

[75] Song P, Liu B, Liang C, Ruan K, Qiu H (2021). Lightweight, flexible cellulose-derived carbon aerogel@reduced graphene oxide/PDMS composites with outstanding EMI shielding performances and excellent thermal conductivities. Nano-Micro Lett..

[76] Yang M, Yang Z, Lv C, Wang Z, Lu Z (2022). Electrospun bifunctional MXene-based electronic skins with high performance electromagnetic shielding and pressure sensing. Compos. Sci. Technol..

[77] Song P, Ma Z, Qiu H, Ru Y, Gu J (2022). High-efficiency electromagnetic interference shielding of rGO@FeNi/epoxy composites with regular honeycomb structures. Nano-Micro Lett..

[78] Chen Y, Liu Y, Li Y, Qi H (2021). Highly sensitive, flexible, stable, and hydrophobic biofoam based on wheat flour for multifunctional sensor and adjustable EMI shielding applications. ACS Appl. Mater. Interfaces.

[79] Cai J-H, Li J, Chen X-D, Wang M (2020). Multifunctional polydimethylsiloxane foam with multi-walled carbon nanotube and thermo-expandable microsphere for temperature sensing, microwave shielding and piezoresistive sensor. Chem. Eng. J..

[80] Gu H, Xu Y, Shen Y, Zhu P, Zhao T (2020). Versatile biomass carbon foams for fast oil–water separation, flexible pressure-strain sensors, and electromagnetic interference shielding. Ind. Eng. Chem. Res..

