# Supplementary material for: Highly Ordered Thermoplastic Polyurethane/Aramid Nanofiber Conductive Foams Modulated by Kevlar Polyanion for Piezoresistive Sensing and Electromagnetic Interference Shielding
Source: Nanomicro Lett. 2023 Apr 7;15:88. doi: 10.1007/s40820-023-01062-0 (PMC10082146; doi:10.1007/s40820-023-01062-0)
Supplement: Supplementary file 1 — Supplementary file1 (PDF 982 kb) [file 40820_2023_1062_MOESM1_ESM.pdf]

Supporting Information for

Highly Ordered Thermoplastic Polyurethane/Aramid Nanofiber Conductive Foams Modulated by Kevlar Polyanion for Piezoresistive Sensing and Electromagnetic Interference Shielding

Kunpeng Qian[1, 2], Jianyu Zhou[2], Miao Miao[2], Hongmin Wu[2], Sineenat Thaiboonrod[3], Jianhui Fang[2], Xin Feng[1, 2, *]

[1] School of Materials Sciences and Engineering, Shanghai University, Shanghai 200444, P. R. China

[2] Research Center of Nano Science and Technology, Shanghai University, Shanghai 200444, P. R. China

[3] National Nanotechnology Center (NANOTEC), National Science and Technology Development Agency (NSTDA), Thailand Science Park, Pathum Thani 12120, Thailand

*Corresponding author. E-mail: fengxin@shu.edu.cn (X. Feng)

Supplementary Figures

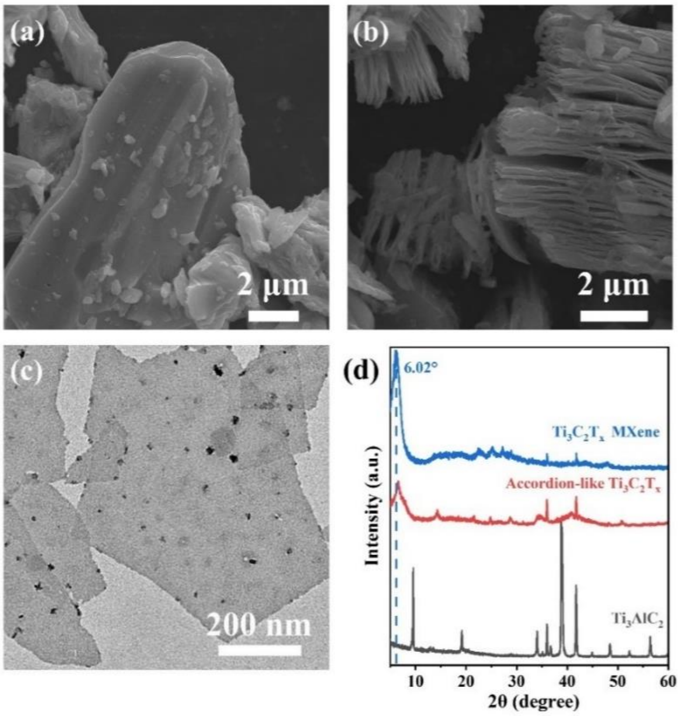

Fig. S1 **a** SEM images of Ti_3AlC_2 bulk and **b** accordion-like $Ti_3C_2T_x$. **c** TEM image of $Ti_3C_2T_x$ MXene. **d** XRD pattern of Ti_3AlC_2, accordion-like $Ti_3C_2T_x$ and $Ti_3C_2T_x$ MXene

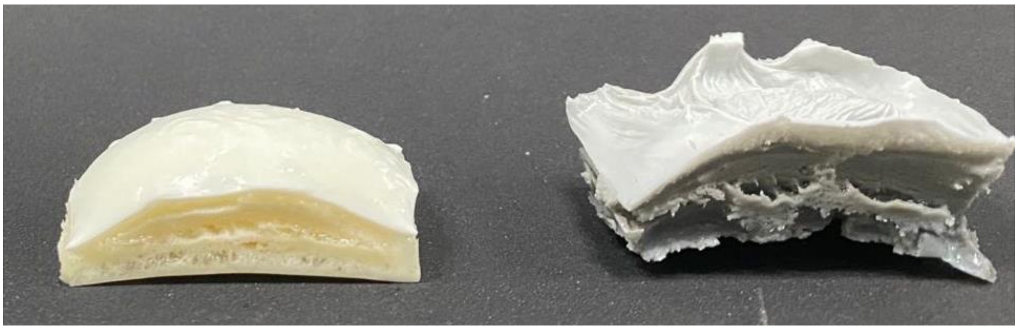

Fig. S2 The visible structural collapse of PA (left) and PAM (right) foam obtained from rectangular groove without freezing pretreatment

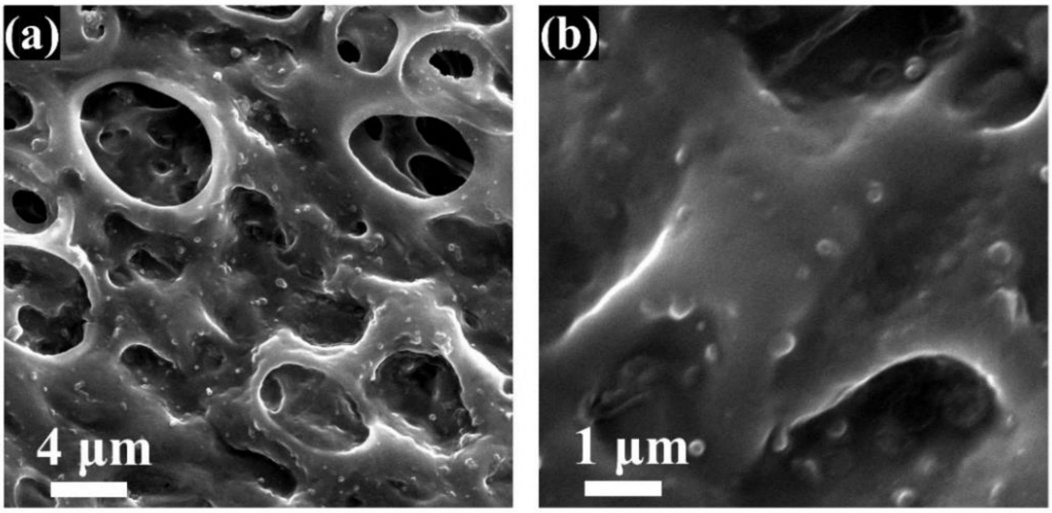

Fig. S3 a SEM images and **b** magnified SEM of PAM foam with Ag NPs anchored

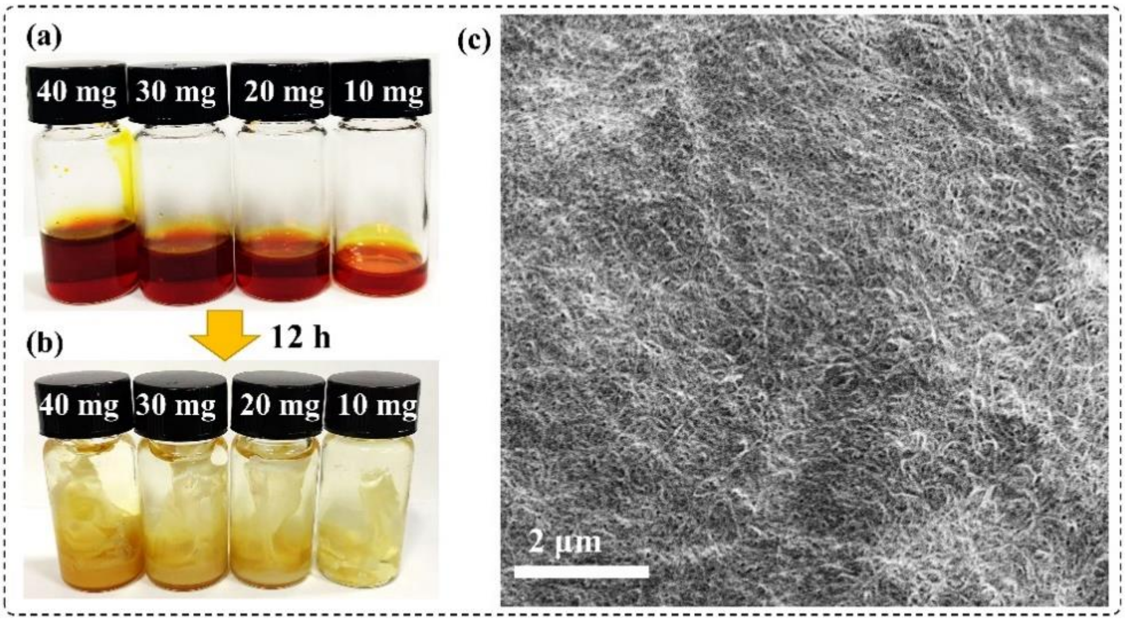

Fig. S4 Digital pictures of **a** Kevlar polyanionic chains solution with different content and **b** precipitated solid of ANF in DI water by protonation after 12 h, correspondingly. **c** SEM image of ANF

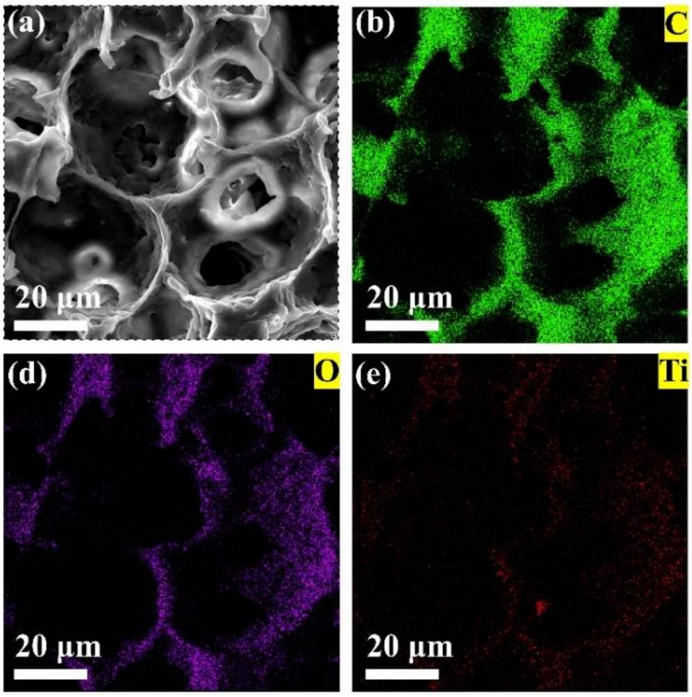

(f)

Elements	Weight (%)
C	56.66
O	29.90
N	13.15
Ti	0.29

Fig. S5 a-e SEM image of PM foam and the corresponding elemental mapping images of C, N, O, Ti. **f** Elements content of C, N, O, Ti

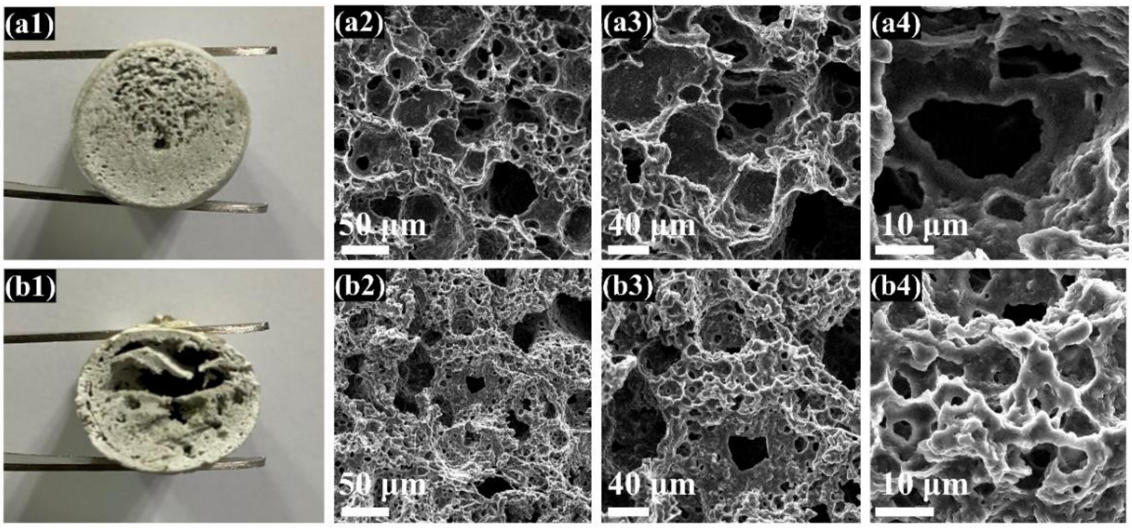

Fig. S6 SEM images of **a** PAM20 and **b** PAM26

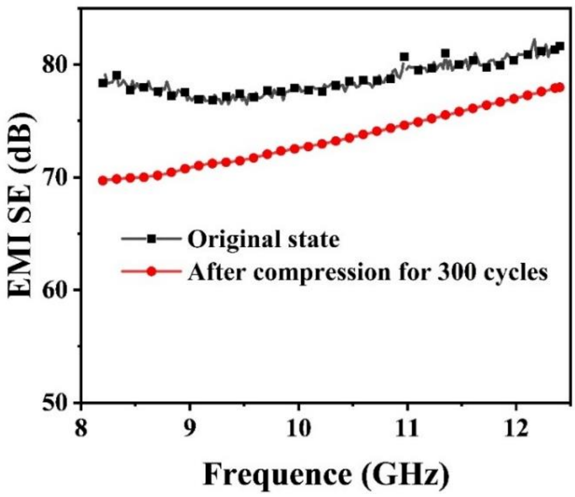

Fig. S7 EMI SE of PAM13-$Cu_{2.0}$ foam before and after 300 compression cycles

The influence of stoichiometry and incorporation of ANF and $Ti_3C_2T_x$ MXene was further analysed in terms of degree of phase separation (DPS). They can be determined based on the peak intensities at 1733 and 1704 cm^{-1} for the free and bonded C=O groups the equations given below:

$$DPS = \frac{C_{bonded}}{C_{bonded} + C_{free}} = \frac{\left(\frac{A_{1704}}{A_{1733}}\right)}{\left(\frac{A_{1704}}{A_{1733}}\right) + 1} = \frac{R}{R+1}$$

Where R represents the carbonyl hydrogen bonding index.

Table S1 R and DSP of TPU, PAM, PAM7, PAM13

**Sample name**	***R***	**DSP**
**TPU**	0.5686	0.3624
**PAM**	0.6114	0.3794
**PAM7**	0.6213	0.3832
**PAM13**	0.6885	0.4077

Table S2 EMI SE, absolute EMI SE (SSE/t), and other physical parameters of PAM and PAM-Cu foam

**Samples**	**EMI SE (dB)**	**Conductivity (S cm^{-1})**	**Density (g cm^{-3})**	**Thickness (mm)**	**SSE/t (dB cm^2 g^{-1})**	**Mass of Cu NPs (mg)**
PAM	0.36	0	0.464	5	1.55	0.0
PAM-$Cu_{0.5}$	21.735	2.28	0.529	5	82.17	90.4
PAM-$Cu_{1.0}$	36.61	9.27	0.548	5	133.61	153.3
PAM-$Cu_{1.5}$	61.54	150.05	0.621	5	198.20	262.1
PAM-$Cu_{2.0}$	79.085	180.00	0.679	5	232.95	292.7

Table S3 EMI SE of different EMI shielding materials in X band

**Filler**	**Matrix**	**Content (wt%)**	**Density ($g\ cm^{-3}$)**	**Thickness (mm)**	**EMI SE (dB)**	**Electrical conductivity ($S\ cm^{-1}$)**	**Refs**
rGO	PEI	10.0	0.29	2.3	13	2.2×10^{-5}	[S1]
rGO	Epoxy	15.0	/	/	21	≈ 0.10	[S2]
rGO	PS	30.0	0.45	2.5	29	1.25×10^{-2}	[S3]
rGO	WPU	7.7	/	2	32	5.1×10^{-2}	[S4]
rGO	WPU	7.5	/	1	34	0.168	[S5]
rGO	PS	7.0	0.26	2.5	45.1	0.435	[S6]
rGO	PU	10.0	0.03	60	57.7	0.6×10^{-3}	[S7]
TGO	PMMA	5.0	0.79	2.4	19	3.11×10^{-2}	[S8]
TGO	PMMA	11.8	/	3.4	30	0.20	[S9]
3D Graphene	PDMS	0.8	0.06	1	20	2	[S10]
Aligned TGO foam	Epoxy	0.8	/	4	32	9.8	[S11]
MWCNT	PLLA	10.0	0.3	2.5	23	3.4×10^{-2}	[S12]
MWCNT	PC	5.0	/	1.85	25	/	[S13]
MWCNT	PP	10.8	/	1	35	≈ 10^{-6}	[S14]
MWCNT	WPU	76.2	0.04	4.5	50	0.446	[S15]
MWCNT	ABS	10.53	/	1.1	50	1.23	[S16]
SWCNT	PU	20.0	/	2	17	2.20×10^{-4}	[S17]
SWCNT	Epoxy	15.0	/	2	28	0.2×10^{-2}	[S18]
SWCNT	Epoxy	20.0	/	4.5	40	2×10^{-2}	[S19]
CNT sponge	Epoxy	2.0	/	2	40	5.16	[S20]
Carbon black	SEBS	15.0	/	5	18	0.22	[S21]
Carbon black	ABS	15.0	0.96	1.1	22	≈ 0.3	[S16]
Carbon nanofiber	PS	15.0	/	/	19	≈ 0.1	[S22]
Carbon nanofiber	ABS	15.0	/	1.1	35	0.66	[S16]
Carbon fiber	PP	16.6	/	3.2	25	0.1	[S23]
Expanded graphite	SEBS	20.0	/	5	12	0.24	[S21]
Graphene nanoplatelets	Epoxy	2.0	/	10	20	2.57×10^{-2}	[S24]
Carbon nanowires/ graphene	PDMS	25.3	0.0971	1.6	36	3.40	[S25]
RGO/Fe_3O_4	PVC	10.0	/	1.8	13	7.70×10^{-6}	[S26]
RGO/Fe_3O_4	PVA	35.0	0.75	0.3	15	/	[S27]

RGO/γ-Fe_2O_3	PVA	53.0	/	0.36	20.3	3×10^{-2}	[S28]
TGO/Fe_3O_4	PS	13.0	/	4	30	0.21	[S29]
Ag Nanowires	PS	21.2	/	0.8	31.85	19	[S30]
Ag Nanowires	PANI	43.4	/	0.04	35	5.30×10^{3}	[S31]
Cu Nanowires	PS	16.0	/	0.2	35	/	[S32]
Cu NPs	TPU	11.3	0.529	5	21.735	2.2765	This work
Cu NPs	TPU	19.2	0.548	5	36.61	9.265	
Cu NPs	TPU	32.8	0.621	5	61.54	150.05	
Cu NPs	TPU	36.6	0.679	5	79.085	180	

/: Unclear or uncalculated value; the numbers of references, which are at the end of the supporting information.

Table S4 Comparison of EMI shielding performance of various composite foams

Filler	Matrix	Content (wt%)	Density (g cm^{-3})	Thickness (mm)	EMI SE (dB)	SSE/t (dB cm^2 g^{-1})	Refs.
Graphene	PMMA	5	0.79	2.4	19	24	[S8]
Graphene	PS	30	0.45	2.5	29	64.4	[S3]
CNT	PS	7	0.56	1.2	19	33	[S33]
Fe_3O_4/Graphene	Paper		0.78	0.3	24	31	[S34]
MCMB/Fe_3O_4	Paper		1.6	2.5	75	47	[S35]
MWCNTs	Phenolic	60.6	0.51	0.14	32.4	63.5	[S36]
MWCNTs	PVDF	15	0.79	2.0	57	76	[S37]
Graphene	PS	7	0.26	2.5	45.1	173	[S6]
Graphene	PEDOT	25	1.04	0.8	70	67.3	[S38]
MWCNT	PC	20	1.13	2.0	43	34.5	[S39]
MWCNT	ABS	15	1.05	1.1	50	47.6	[S16]
MWCNT	PS	20	0.53	2.0	30	57	[S40]
rGO	PI	16	0.28	0.8	21	75	[S41]
G@Fe_3O_4	PEI	10	0.4	2.5	18	42	[S42]
Graphene	PEI	10	0.3	2.3	13	44	[S43]
MWCNT	Epoxy	3	0.33	2.8	7.1	21.3	[S44]
CNT	scPLA	30	0.1	3.7	21.6	216	[S45]
MCMB-MWCNTs	Paper	25	0.26	0.6	56	215	[S46]
RGO	TPU	6.5	0.8	1.8	21.8	16.6	[S47]
CNTs @Fe_3O_4	PMMA	7	0.38	2.5	13.1	50	[S4]
RGO/MWCNTs	PI	8	0.44	0.5	18.2	41	[S48]
MWCNTs	PLLA	10	0.3	2.5	23	77	[S49]
10Ni-CNT /10CNT/PIL	TPU	20	0.33	2.0	69.8	211.5	[S50]
Cu NPs	TPU	36.6	0.679	5	79.085	232.95	This work

Supplementary References (Fig. 9c [S1-S50] and Fig. 9d [S51-S64])

[S1] J. Ling, W. Zhai, W. Feng, B. Shen, J. Zhang, W. Zheng. Facile preparation of lightweight microcellular polyetherimide/graphene composite foams for electromagnetic interference shielding. ACS Appl. Mater. Interfaces **5**, 2677-84 (2013). https://doi.org/10.1021/am303289m

[S2] J. Liang, Y. Wang, Y. Huang, Y. Ma, Z. Liu, J. Cai, C. Zhang, H. Gao, Y. Chen. Electromagnetic interference shielding of graphene/epoxy composites. Carbon **47**, 922-5 (2009). https://doi.org/10.1016/j.carbon.2008.12.038

[S3] D.-X. Yan, P.-G. Ren, H. Pang, Q. Fu, M.-B. Yang, Z.-M. Li. Efficient electromagnetic interference shielding of lightweight graphene/polystyrene composite. J. Mater. Chem. **22**, (2012). https://doi.org/10.1039/c2jm32692b

[S4] S.-T. Hsiao, C.-C.M. Ma, H.-W. Tien, W.-H. Liao, Y.-S. Wang, S.-M. Li, Y.-C. Huang. Using a non-covalent modification to prepare a high electromagnetic interference shielding performance graphene nanosheet/water-borne polyurethane composite. Carbon **60**, 57-66 (2013). https://doi.org/10.1016/j.carbon.2013.03.056

[S5] S.-T. Hsiao, C.-C.M. Ma, W.-H. Liao, Y.-S. Wang, S.-M. Li, Y.-C. Huang, R.-B. Yang, W.-F. Liang. Lightweight and flexible reduced graphene oxide/water-borne polyurethane composites with high electrical conductivity and excellent electromagnetic interference shielding performance. ACS Appl. Mater. Interfaces **6**, 10667-78 (2014). https://doi.org/10.1021/am502412q

[S6] D.-X. Yan, H. Pang, B. Li, R. Vajtai, L. Xu, P.-G. Ren, J.-H. Wang, Z.-M. Li. Structured Reduced Graphene Oxide/Polymer Composites for Ultra-Efficient Electromagnetic Interference Shielding. Adv. Funct. Mater. **25**, 559-66 (2015). https://doi.org/10.1002/adfm.201403809

[S7] B. Shen, Y. Li, W. Zhai, W. Zheng. Compressible Graphene-Coated Polymer Foams with Ultralow Density for Adjustable Electromagnetic Interference (EMI) Shielding. ACS Appl Mater Interfaces **8**, 8050-7 (2016). https://doi.org/10.1021/acsami.5b11715

[S8] H.B. Zhang, Q. Yan, W.G. Zheng, Z. He, Z.Z. Yu. Tough graphene-polymer microcellular foams for electromagnetic interference shielding. ACS Appl. Mater. Interfaces **3**, 918-24 (2011). https://doi.org/10.1021/am200021v

[S9] H.-B. Zhang, W.-G. Zheng, Q. Yan, Z.-G. Jiang, Z.-Z. Yu. The effect of surface chemistry of graphene on rheological and electrical properties of polymethylmethacrylate composites. Carbon **50**, 5117-25 (2012). https://doi.org/10.1016/j.carbon.2012.06.052

[S10] Z. Chen, C. Xu, C. Ma, W. Ren, H.M. Cheng. Lightweight and flexible graphene foam composites for high-performance electromagnetic interference shielding. Adv. Mater. **25**, 1296-300 (2013).

https://doi.org/10.1002/adma.201204196

[S11] X.H. Li, X. Li, K.N. Liao, P. Min, T. Liu, A. Dasari, Z.Z. Yu. Thermally Annealed Anisotropic Graphene Aerogels and Their Electrically Conductive Epoxy Composites with Excellent Electromagnetic Interference Shielding Efficiencies. ACS Appl. Mater. Interfaces **8**, 33230-9 (2016). https://doi.org/10.1021/acsami.6b12295

[S12] T. Kuang, L. Chang, F. Chen, Y. Sheng, D. Fu, X. Peng. Facile preparation of lightweight high-strength biodegradable polymer/multi-walled carbon nanotubes nanocomposite foams for electromagnetic interference shielding. Carbon **105**, 305-13 (2016). https://doi.org/10.1016/j.carbon.2016.04.052

[S13] M. Arjmand, M. Mahmoodi, G.A. Gelves, S. Park, U. Sundararaj. Electrical and electromagnetic interference shielding properties of flow-induced oriented carbon nanotubes in polycarbonate. Carbon **49**, 3430-40 (2011). https://doi.org/10.1016/j.carbon.2011.04.039

[S14] 14. M.H. Al-Saleh, U. Sundararaj. Electromagnetic interference shielding mechanisms of CNT/polymer composites. Carbon **47**, 1738-46 (2009). https://doi.org/10.1016/j.carbon.2009.02.030

[S15] Z. Zeng, H. Jin, M. Chen, W. Li, L. Zhou, Z. Zhang. Lightweight and Anisotropic Porous MWCNT/WPU Composites for Ultrahigh Performance Electromagnetic Interference Shielding. Adv. Funct. Mater. **26**, 303-10 (2016). https://doi.org/10.1002/adfm.201503579

[S16] M.H. Al-Saleh, W.H. Saadeh, U. Sundararaj. EMI shielding effectiveness of carbon based nanostructured polymeric materials: A comparative study. Carbon **60**, 146-56 (2013). https://doi.org/10.1016/j.carbon.2013.04.008

[S17] Z. Liu, G. Bai, Y. Huang, Y. Ma, F. Du, F. Li, T. Guo, Y. Chen. Reflection and absorption contributions to the electromagnetic interference shielding of single-walled carbon nanotube/polyurethane composites. Carbon **45**, 821-7 (2007). https://doi.org/10.1016/j.carbon.2006.11.020

[S18] Y. Yang, M.C. Gupta, K.L. Dudley, R.W.J.N.l. Lawrence. Novel carbon nanotube− polystyrene foam composites for electromagnetic interference shielding. Nano Lett. **5**, 2131-4 (2005). https://doi.org/10.1021/nl051375r

[S19] Y. Huang, N. Li, Y. Ma, F. Du, F. Li, X. He, X. Lin, H. Gao, Y. Chen. The influence of single-walled carbon nanotube structure on the electromagnetic interference shielding efficiency of its epoxy composites. Carbon **45**, 1614-21 (2007). https://doi.org/10.1016/j.carbon.2007.04.016

[S20] Y. Chen, H.-B. Zhang, Y. Yang, M. Wang, A. Cao, Z.-Z. Yu. High-Performance Epoxy Nanocomposites Reinforced with Three-Dimensional Carbon Nanotube Sponge for Electromagnetic Interference Shielding. Adv. Funct. Mater. **26**, 447-55 (2016). https://doi.org/10.1002/adfm.201503782

[S21] S. Kuester, C. Merlini, G.M.O. Barra, J.C. Ferreira, A. Lucas, A.C. de Souza,

B.G. Soares. Processing and characterization of conductive composites based on poly(styrene-b-ethylene-ran-butylene-b-styrene) (SEBS) and carbon additives: A comparative study of expanded graphite and carbon black. Composites, Part B. **84**, 236-47 (2016). https://doi.org/10.1016/j.compositesb.2015.09.001

[S22] Y. Yang, M.C. Gupta, K.L. Dudley, R.W. Lawrence. Conductive Carbon Nanofiber-Polymer Foam Structures. Adv. Mater. **17**, 1999-2003 (2005). https://doi.org/10.1002/adma.200500615

[S23] A. Ameli, P.U. Jung, C.B. Park. Electrical properties and electromagnetic interference shielding effectiveness of polypropylene/carbon fiber composite foams. Carbon **60**, 379-91 (2013). https://doi.org/10.1016/j.carbon.2013.04.050

[S24] G. De Bellis, A. Tamburrano, A. Dinescu, M.L. Santarelli, M.S. Sarto. Electromagnetic properties of composites containing graphite nanoplatelets at radio frequency. Carbon **49**, 4291-300 (2011). https://doi.org/10.1016/j.carbon.2011.06.008

[S25] L. Kong, X. Yin, M. Han, X. Yuan, Z. Hou, F. Ye, L. Zhang, L. Cheng, Z. Xu, J. Huang. Macroscopic bioinspired graphene sponge modified with in-situ grown carbon nanowires and its electromagnetic properties. Carbon **111**, 94-102 (2017). https://doi.org/10.1016/j.carbon.2016.09.066

[S26] K. Yao, J. Gong, N. Tian, Y. Lin, X. Wen, Z. Jiang, H. Na, T. Tang. Flammability properties and electromagnetic interference shielding of PVC/graphene composites containing Fe_3O_4 nanoparticles. RSC Adv. **5**, 31910-9 (2015). https://doi.org/10.1039/c5ra01046b

[S27] B.V. Rao, P. Yadav, R. Aepuru, H.S. Panda, S. Ogale, S.N. Kale. Single-layer graphene-assembled 3D porous carbon composites with PVA and Fe_3O_4 nano-fillers: an interface-mediated superior dielectric and EMI shielding performance. Phys. Chem. Chem. Phys. **17**, 18353-63 (2015). https://doi.org/10.1039/c5cp02476e

[S28] B. Yuan, C. Bao, X. Qian, L. Song, Q. Tai, K.M. Liew, Y. Hu. Design of artificial nacre-like hybrid films as shielding to mitigate electromagnetic pollution. Carbon. **75**, 178-89 (2014). https://doi.org/10.1016/j.carbon.2014.03.051

[S29] Y. Chen, Y. Wang, H.-B. Zhang, X. Li, C.-X. Gui, Z.-Z. Yu. Enhanced electromagnetic interference shielding efficiency of polystyrene/graphene composites with magnetic Fe_3O_4 nanoparticles. Carbon **82**, 67-76 (2015). https://doi.org/10.1016/j.carbon.2014.10.031

[S30] M. Arjmand, A.A. Moud, Y. Li, U.J.R.A. Sundararaj. Outstanding electromagnetic interference shielding of silver nanowires: comparison with carbon nanotubes. **5**, 56590-8 (2015). RSC Adv.

https://doi.org/10.1039/C5RA01046B

[S31] F. Fang, Y.-Q. Li, H.-M. Xiao, N. Hu, S.-Y. Fu. Layer-structured silver nanowire/polyaniline composite film as a high performance X-band EMI shielding material. J. Mater. Chem. C. **4**, 4193-203 (2016). https://doi.org/10.1039/c5tc04406e

[S32] M.H. Al-Saleh, G.A. Gelves, U. Sundararaj. Copper nanowire/polystyrene nanocomposites: Lower percolation threshold and higher EMI shielding. Composites, Part A. **42**, 92-7 (2011). https://doi.org/10.1016/j.compositesa.2010.10.003

[S33] Y. Yang, M.C. Gupta, K.L. Dudley, R.W.J.N.L. Lawrence. Novel Carbon Nanotube−Polystyrene Foam Composites for Electromagnetic Interference Shielding. Nano lett. **5**, 2131-2134 (2005). https://doi.org/10.1021/nl051375r

[S34] W.-L. Song, X.-T. Guan, L.-Z. Fan, W.-Q. Cao, C.-Y. Wang, Q.-L. Zhao, M.-S. Cao. Magnetic and conductive graphene papers toward thin layers of effective electromagnetic shielding. J. Mater. Chem. A. **3**, 2097-107 (2015). https://doi.org/10.1039/c4ta05939e

[S35] R. Dhawan, S. Kumari, R. Kumar, S.K. Dhawan, S.R. Dhakate. Mesocarbon microsphere composites with Fe3O4 nanoparticles for outstanding electromagnetic interference shielding effectiveness. RSC Adv. **5**, 43279-89 (2015). https://doi.org/10.1039/c5ra03332b

[S36] S. Teotia, B.P. Singh, I. Elizabeth, V.N. Singh, R. Ravikumar, A.P. Singh, S. Gopukumar, S.K. Dhawan, A. Srivastava, R.B. Mathur. Multifunctional, robust, light-weight, free-standing MWCNT/phenolic composite paper as anodes for lithium ion batteries and EMI shielding material. RSC Adv. **4**, 33168-74 (2014). https://doi.org/10.1039/c4ra04183f

[S37] H. Wang, K. Zheng, X. Zhang, X. Ding, Z. Zhang, C. Bao, L. Guo, L. Chen, X. Tian. 3D network porous polymeric composites with outstanding electromagnetic interference shielding. Compos. Sci. Technol. **125**, 22-9 (2016). https://doi.org/10.1016/j.compscitech.2016.01.007

[S38] N. Agnihotri, K. Chakrabarti, A. De. Highly efficient electromagnetic interference shielding using graphite nanoplatelet/poly(3,4-ethylenedioxythiophene)–poly(styrenesulfonate) composites with enhanced thermal conductivity. RSC Adv. **5**, 43765-71 (2015). https://doi.org/10.1039/c4ra15674a

[S39] S. Pande, A. Chaudhary, D. Patel, B.P. Singh, R.B. Mathur. Mechanical and electrical properties of multiwall carbon nanotube/polycarbonate composites for electrostatic discharge and electromagnetic interference shielding applications. RSC Adv. **4**, (2014). https://doi.org/10.1039/c3ra47387b

[S40] M. Arjmand, T. Apperley, M. Okoniewski, U. Sundararaj. Comparative study of electromagnetic interference shielding properties of injection molded versus

compression molded multi-walled carbon nanotube/polystyrene composites. Carbon. **50**, 5126-34 (2012). https://doi.org/10.1016/j.carbon.2012.06.053

[S41] Z. Zeng, M. Chen, H. Jin, W. Li, X. Xue, L. Zhou, Y. Pei, H. Zhang, Z. Zhang. Thin and flexible multi-walled carbon nanotube/waterborne polyurethane composites with high-performance electromagnetic interference shielding. Carbon. **96**, 768-77 (2016). https://doi.org/10.1016/j.carbon.2015.10.004

[S42] X. Lv, Y. Tang, Q. Tian, Y. Wang, T. Ding. Ultra-stretchable membrane with high electrical and thermal conductivity via electrospinning and in-situ nanosilver deposition. Compos. Sci. Technol. **200**, 768-77 (2020). https://doi.org/10.1016/j.compscitech.2020.108414

[S43] J. Chen, Y. Zhu, Z. Guo, A.G. Nasibulin. Recent Progress on Thermo-electrical Properties of Conductive Polymer Composites and Their Application in Temperature Sensors. Eng. Sci. (2020). https://doi.org/10.30919/es8d1129

[S44] L. Wang, P. Song, C.T. Lin, J. Kong, J. Gu. 3D Shapeable, superior electrically conductive cellulose nanofibers/$Ti_3C_2T_x$ MXene aerogels/epoxy nanocomposites for promising EMI shielding. Research **2020**, 4093732 (2020). https://doi.org/10.34133/2020/4093732

[S45] Y. Gu, R.M. Dorin, U. Wiesner. Asymmetric organic-inorganic hybrid membrane formation via block copolymer-nanoparticle co-assembly. Nano Lett. **13**, 5323-8 (2013). https://doi.org/10.1021/nl402829p

[S46] J. Zhao, G. Luo, J. Wu, H. Xia. Preparation of microporous silicone rubber membrane with tunable pore size via solvent evaporation-induced phase separation. ACS Appl. Mater. Interfaces **5**, 2040-6 (2013). https://doi.org/10.1021/am302929c

[S47] X. Ma, B. Shen, L. Zhang, Y. Liu, W. Zhai et al., Porous superhydrophobic polymer/carbon composites for lightweight and self-cleaning EMI shielding application. Compos. Sci. Technol. **158**, 86-93 (2018). https://doi.org/10.1016/j.compscitech.2018.02.006

[S48] S. Ganguly, S. Ghosh, P. Das, T.K. Das, S.K. Ghosh et al., Poly(N-vinylpyrrolidone)-stabilized colloidal graphene-reinforced poly(ethylene-co-methyl acrylate) to mitigate electromagnetic radiation pollution. Polym. Bull. **77**, 2923-43 (2019). https://doi.org/10.1007/s00289-019-02892-y

[S49] D.R. Dreyer, S. Park, C.W. Bielawski, R.S. Ruoff. The chemistry of graphene oxide. Chem. Soc. Rev. **39**, 228-40 (2010). https://doi.org/10.1039/b917103g

[S50] G. Sang, P. Xu, T. Yan, V. Murugadoss, N. Naik et al., Interface engineered microcellular magnetic conductive polyurethane nanocomposite foams for electromagnetic interference shielding. Nano-Micro Lett. **13**, 153 (2021). https://doi.org/10.1007/s40820-021-00677-5

[S51] J. Zhai, Y. Zhang, C. Cui, A. Li, W. Wang et al., Flexible waterborne polyurethane/cellulose nanocrystal composite aerogels by integrating graphene

and carbon nanotubes for a highly sensitive pressure sensor. ACS Sustainable Chem. Eng. **9**, 14029-39 (2021). https://doi.org/10.1021/acssuschemeng.1c03068

[S52] A. Tewari, S. Gandla, S. Bohm, C.R. McNeill, D. Gupta, Highly exfoliated MWNT-rGO ink-wrapped polyurethane foam for piezoresistive pressure sensor applications. ACS Appl. Mater. Interfaces **10**, 5185-95 (2018). https://doi.org/10.1021/acsami.7b15252

[S53] L. Pu, Y. Liu, L. Li, C. Zhang, P. Ma et al., Polyimide nanofiber-reinforced $Ti_3C_2T_x$ aerogel with "lamella-pillar" microporosity for high-performance piezoresistive strain sensing and electromagnetic wave absorption. ACS Appl. Mater. Interfaces **13**, 47134-46 (2021). https://doi.org/10.1021/acsami.1c13863

[S54] Y. Zhai, Y. Yu, K. Zhou, Z. Yun, W. Huang et al., Flexible and wearable carbon black/thermoplastic polyurethane foam with a pinnate-veined aligned porous structure for multifunctional piezoresistive sensors. Chem. Eng. J. **382**, (2020). https://doi.org/10.1016/j.cej.2019.122985

[S55] H.B. Yao, J. Ge, C.F. Wang, X. Wang, W. Hu et al., A flexible and highly pressure-sensitive graphene-polyurethane sponge based on fractured microstructure design. Adv. Mater. **25**, 6692-8 (2013). https://doi.org/10.1002/adma.201303041

[S56] X.P. Li, Y. Li, X. Li, D. Song, P. Min et al., Highly sensitive, reliable and flexible piezoresistive pressure sensors featuring polyurethane sponge coated with MXene sheets. J. Colloid Interface Sci. **542**, 54-62 (2019). https://doi.org/10.1016/j.jcis.2019.01.123

[S57] Z. Chen, Y. Hu, H. Zhuo, L. Liu, S. Jing et al., Compressible, elastic, and pressure-sensitive carbon aerogels derived from 2d titanium carbide nanosheets and bacterial cellulose for wearable sensors. Chem. Mater. **31**, 3301-12 (2019). https://doi.org/10.1021/acs.chemmater.9b00259

[S58] J. Xiao, Y. Tan, Y. Song, Q. Zheng, A flyweight and superelastic graphene aerogel as a high-capacity adsorbent and highly sensitive pressure sensor. J. Mater. Chem. A **6**, 9074-80 (2018). https://doi.org/10.1039/c7ta11348j

[S59] Y. Ma, Y. Yue, H. Zhang, F. Cheng, W. Zhao et al., 3D synergistical mxene/reduced graphene oxide aerogel for a piezoresistive sensor. ACS Nano **12**, 3209-16 (2018). https://doi.org/10.1021/acsnano.7b06909

[S60] I. You, S.-E. Choi, H. Hwang, S.W. Han, J.W. Kim et al., E-skin tactile sensor matrix pixelated by position-registered conductive microparticles creating pressure-sensitive selectors. Adv. Funct. Mater. **28**, (2018). https://doi.org/10.1002/adfm.201801858

[S61] G.Y. Bae, S.W. Pak, D. Kim, G. Lee, H. Kim do et al., Linearly and highly pressure-sensitive electronic skin based on a bioinspired hierarchical structural array. Adv. Mater. **28**, 5300-6 (2016). https://doi.org/10.1002/adma.201600408

[S62] X. Wu, Y. Han, X. Zhang, Z. Zhou, C. Lu, Large-area compliant, low-cost, and versatile pressure-sensing platform based on microcrack-designed carbon black@polyurethane sponge for human-machine interfacing. Adv. Funct. Mater. **26**, 6246-56 (2016). https://doi.org/10.1002/adfm.201601995

[S63] Y. Si, X. Wang, C. Yan, L. Yang, J. Yu et al., Ultralight biomass-derived carbonaceous nanofibrous aerogels with superelasticity and high pressure-sensitivity. Adv. Mater. **28**, 9512-8 (2016). https://doi.org/10.1002/adma.201603143

[S64] B. Su, S. Gong, Z. Ma, L.W. Yap, W. Cheng. Mimosa-inspired design of a flexible pressure sensor with touch sensitivity. Small **11**, 1886-91 (2015). https://doi.org/10.1002/smll.201403036
